# Acidosis enhances the self-renewal and mitochondrial respiration of stem cell-like glioma cells through CYP24A1-mediated reduction of vitamin D

**DOI:** 10.1038/s41419-018-1242-1

**Published:** 2019-01-10

**Authors:** Peishan Hu, Shanshan Li, Ningyu Tian, Fan Wu, Yan Hu, Dengke Li, Yingjiao Qi, Zhizhong Wei, Qunfang Wei, Yanchao Li, Bin Yin, Tao Jiang, Jiangang Yuan, Boqin Qiang, Wei Han, Xiaozhong Peng

**Affiliations:** 1 State Key Laboratory of Medical Molecular Biology, Department of Molecular Biology and Biochemistry, Institute of Basic Medical Sciences, Medical Primate Research Center, Neuroscience Center, Chinese Academy of Medical Sciences, School of Basic Medicine Peking Union Medical College, 100005 Beijing, China; 2 Department of Molecular Neuropathology, Beijing Neurosurgical Institute, Capital Medical University, Beijing, China; 3 Department of Neurosurgery, Beijing Tiantan Hospital, Capital Medical University, Beijing, China; 4 Institute of Medical Biology, Chinese Academy of Medical Sciences, Peking Union Medical College, Kunming, China

## Abstract

Acidosis is a significant feature of the tumor microenvironment in glioma, and it is closely related to multiple biological functions of cancer stem cells. Here, we found that the self-renewal ability, the mitochondrial activity and ATP production were elevated in stem cell-like glioma cells (SLCs) under acidic microenvironment, which promoted and maintained the stemness of SLCs. Under acidosis, 25-hydroxy vitamin D_3_-24-hydroxylase (CYP24A1) was upregulated and catalyzed the fast degradation of 1α,25(OH)_2_D_3_. We further revealed that the active form of vitamin D (1α,25(OH)_2_D_3_) could inhibit the expression of stemness markers, attenuate acidosis-induced increase of self-renewal ability and mitochondrial respiration in stem cell-like glioma cells. Our study indicates that the acidosis–CYP24A1–vitamin D pathway may be a key regulator of the cancer stem cell phenotype in malignant glioma and point out the potential value for the utilization of vitamin D to target cancer stem cells and to restrain the growth of malignant glioma in the future.

## Introduction

Due to the character of rapid and infiltrative growth, high recurrence, as well as the resistance to radiation and chemotherapy, the prognosis of glioma remains very poor^1^. In recent years, evidence shows that these features are closely associated with the existence of glioma stem cells (GSCs). A small percentage of tumor cells in tumor tissue have the character of stem cells, which are referred to as cancer stem cells (CSCs)^2^, which are known as the root of tumor growth and recurrence. The development and progression of glioma are regulated by various factors, including stem cell pathways, metabolic conversion, epigenetic modification, copy number variation, gene fusion, somatic mutation, and tumor microenvironment^3^. Tumor microenvironment plays an important role in the stem cell fate decision, and eventually results in the poor treatment outcome^4^. In addition, it has been reported that normalizing the tumor microenvironment can improve the curative effect^5^.

The main features of microenvironment in glioma is low pH value. It has been reported that the pH value is ~7.1 in the normal brain tissue, while in glioma tissues the pH value is about 6.8^6^. Low pH is thought to be the driver of tumor progression and treatment resistance^5,7,8^. Furthermore, low pH are the determinant factors of tumor cell metabolism phenotype, which can provide the basic requirements of the tumor cells by changing the core cell metabolic phenotype and making cancer cells to reach its groove^9–12^.

The study of acidic environment in glioma began in 2001, evidence found that acid environment can increase the transcription of vascular endothelial growth factor (VEGF) in brain glioma cells^13^, further revealed that the acid environment induced the expression of VEGF through activation of the Ras and ERK1/2 MAPK-signaling pathways^14^. The acidic environments promoted and maintained glioma stem cell phenotype through inducing the expression of HIF2 alpha and HIF target genes^15^. Furthermore, Filatova et al. found that acidic environment increased the expression of hypoxia inducible factor (HIF) by heat shock protein 90 (HSP90), rather than PDH/VHL dependence pathway, in order to maintain the stemness of glioma cells^16^. Studies on the metabolism of GSCs also have made great progress. It is reported that GSCs maintain their demands for energy and biological macromolecular materials mainly through oxidative phosphorylation in mitochondria. Compared with the highly differentiated glioma cells, GSCs consume less glucose, maintain high levels of ATP and mitochondrial respiratory reserve capacity^17^. Likewise, mitochondrial dynamic regulates the biology characteristics of glioma stem cell, loss of Dynein protein 1 (DRP1) inhibits the proliferation, self-renewal, and tumor formation of glioma stem cell^18^. At the same time, latest study showed that acetyl coenzyme A was mainly provided by the fatty acid oxidation in a variety of solid tumors cells when the cells were in the acidic conditions, which changed the glucose metabolism in general condition and maintained tricarboxylic acid cycle and respiration of tumor cells. These results suggested that tumor cells can sustain their survival by switching their major metabolic pathways when they are in acid condition^19^.

As an important feature of the microenvironment of glioma, low pH regulates the angiogenesis, invasion, and resistance to chemotherapy of glioma. But the mechanism of adaptation in acidic environment of glioma cells and their metabolic changes induced by acidic environment are still unclear. In this study, we found that the self-renewal ability and mitochondrial respiration were elevated in stem cell-like glioma cells (SLCs) in acidic microenvironment. CYP24A1 is expressed in the inner membrane of mitochondria, served as 25-hydroxyvitamin D_3_-24-hydroxylase and degraded the active hormone 1α,25-dihydroxyvitamin D_3_^20^. CYP24A1 was accounted for the stemness and metabolism changes of SLCs, mainly through the fast degradation of 1α,25-dihydroxyvitamin D_3_. Our study suggested that it might be a potential treatment strategy to target CYP24A1 for inhibiting the growth of malignant glioma under acidic microenvironment.

## Methods and materials

### Glioma samples and cell lines

Fresh glioma samples were obtained from Beijing Tiantan Hospital and Beijing Sanbo Brain Hospital. Human glioma cell lines (U87MG and T98G) were purchased from the American Type Culture Collection (ATCC) (Manassas, VA). U251 cells were purchased from the Cell Center of Peking Union Medical College. Four human normal astrocyte cell lines (HA, HA-sp, NHA, and HA-c) were purchased from ScienCell (Carlsbad, CA).

### Cell culture and treatments

The U87MG and T98G cells were cultured in modified Eagle’s medium (MEM), and the U251 cells were cultured in Dulbecco’s modified Eagle’s medium (DMEM). Both media were supplemented with 10% fetal bovine serum (FBS) (HyClone, Logan City, UT), 100 U/mL penicillin (Life Science), and 100 U/mL streptomycin (Life Science). The normal human astrocyte cells (HA, HA-c, NHA, and HA-sp) were cultured in astrocyte medium (ScienCell, Carlsbad, CA).

Neurosphere formation culture and primary stem cell-like glioma cell culture from glioma specimens were performed as described previously^21^.

For different pH treatment, 25 mM HEPES (Sigma, USA) was added into SFM, 1 M HCl or 1 M NaOH was used to titrate SFM until the pH value reached 7.4, 6.8, 6.7, 6.6, and 6.5. Then incubate for 24 h and retitrate to the corresponding pH value^22^.

### Limiting dilution assay and neurosphere formation assay

The limiting dilution assay was performed as described previously^23^. In brief, the sphere cells were plated in 96-well plates in 100 μL SFM culture medium. Final cell densities ranged from 500 to 3 cells/well in 100 μL volumes. After 12 days, the percentage of wells that are not containing spheres (diameter ≥ 50 μm) for each cell plating density was calculated.

The neurosphere-forming assay was performed as described previously^23^. Cells were diluted to 1000–5000 cells per 100 μL. The cells were allowed to grow for 6–8 days, with 50 μL fresh SFM added at day 3 or 4. Then, diameter of the neurospheres ≥ 50 μm in each well were counted under a microscope.

### Immunoblotting

Immunoblotting was performed as described previously^24^. β-actin (Sigma, St. Louis, MO) was used as inter control. The antibodies used included anti-CYP24A1 (Abcam, UK), anti-GFAP (Abcam, UK), anti-Tuj1 (Sigma, USA), anti-Sox2 (Abcam, UK; Abclonal, Beijing, China), anti-CD133 (Miltenyi Biotec, Germany; Abclonal, Beijing, China), anti-Nestin (Abclonal, Beijing, China), anti-Oct4 (Abclonal, Beijing, China), anti-CNPase (Abclonal, Beijing, China), and anti-β-actin (Sigma, USA).

### Immunostaining

For staining the SLC spheres and differentiated SLCs, the cells were fixed with 4% paraformaldehyde, permeabilized by 0.3% Triton X-100 (Sigma), and incubated with the indicated first antibodies and fluorescence-conjugated secondary antibodies (Molecular Probes, Life Technologies, CA) and stained with 4′,6-diamidino-2-phenylindole (DAPI, Sigma) for nuclear staining. The stained cells were observed with a fluorescence microscope (Olympus, Japan). The antibodies used included anti-CYP24A1 (Abcam, UK) and anti-CAIX (Abcam, UK). Fluorescence-conjugated secondary antibodies were from Life Technologies (CA, USA).

### Mitochondrial respiration analysis

The oxygen-consumption rates (OCR) of glioma cells and SLCs were measured using the Seahorse XF Extracellular Flux Analyzer (Seahorse Bioscience). Twenty-four-well plates (Seahorse Bioscience) were coated with 50 μL poly-d-lysine (10 μg/mL) for 2 h then coated with laminin (10 μg/mL) overnight. The next day washed the wells twice with saline, and 10,000–100,000 cells were plated per well. Three metabolic inhibitors were injected sequentially as specific time points: oligomycin (1 μM), followed by FCCP (0.75 μM), followed by a combination of rotenone and antimycinA (0.5 μM). Basal OCR were measured using the Seahorse XF24 plate reader. Several parameters of oxygen consumption were analyzed as previously reported^25^.

### Quantitative real-time RT-PCR

Real-time RT-PCR (QRT-PCR) was carried out using SYBR Premix Ex Taq Master Mix and a 2-Step kit (TaKaRa, Dalian, China) as described previously^3^. In brief, total RNA was isolated using TRIzol reagent according to the manufacturer’s instructions (Invitrogen, Carlsbad, CA). 2.0 μg total RNA was used for cDNA synthesis (Transgene, Beijing, China) and 1/10 of the cDNA volume was used for quantitative PCR (TaKaRa, Dalian, China). The PCR amplification was carried out using a CFX96 touch system (BioRad, USA) or an ABI 7500 system (Life Science, USA). The Ct values were normalized to human glyceraldehyde-3-phosphate dehydrogenase (GAPDH) gene. All samples were run in triplicate in each experiment. Each assay was repeated three to four times. All primers were synthesized by Life Technology (Beijing, China) and TSINGKE (Beijing, China).

### LncRNA and mRNA arrays

~10^6^ pH 7.4 or pH 6.8 treated GSC5 cells were digested by accutase (Invitrogen). Total RNA from cells were lysed using TRIzol reagent (Invitrogen). The RNA integrity was measured at an Agilent Bioanalyzer 2100 instrument (Agilent Technologies, Santa Clara, CA). The qualified total RNA was further purified using NucleoSpin^®^ RNA clean-up Kit. Total RNA was amplified and labeled with a Low Input Quick Amp Labeling Kit (Agilent Technologies), per the manufacturer’s instructions. The labeled cRNA was purified with an NucleoSpin^®^ RNA clean-up Kit, and 10 μg RNA was used for hybridization on the Genechip Agilent Human lncRNA 4*180K array (Agilent Technologies). The slides were scanned in an Agilent Microarray Scanner G2565CA (Agilent Technologies) under the default settings. Quantile algorithm (Gene Spring Software, Agilent Technologies) was used for the raw data normalization.

### 1α,25(OH)_2_D_3_ quantification

For the quantification of 1α,25(OH)_2_D_3_ in SLCs, 1 μM 25(OH)D_3_ (Sigma, dissolved in methanol) was added into the cell culture medium. After 12 h, GSC2 cells were collected under yellow light, 5 × 10^7^ cells were quenched by directly adding 500 μL CH_3_OH, and then extracted by adding 1 mL methyl tert-butyl ether (MTBE). The extraction solvent is equivalent to MTBE–CH_3_OH (2:1, V/V). The cells were vortexed for 5 min (2500 rpm) and transferred to a microcentrifuge tube and snap-frozen in liquid nitrogen. The cells were then thawed and vortexed for 60 s (2500 rpm), the freeze–thaw process was repeated for two more cycles, and the cells were then vortexed for 5 min (2500 rpm) and centrifuged at 12,000 rpm for 10 min. The supernatant was transferred to a microcentrifuge tube and dried by using a centrifugal evaporator at 35 °C and 150 μL MeOH–IPA–H_2_O (65:30:5, V/V/V) was added to the residue. The samples were vortexed for 5 min (2500 rpm) and centrifuged at 12,000 rpm for 10 min, and the supernatant was then transferred to autosampler vials for analysis. 1α,25(OH)_2_D_3_ was quantified by a UPLC–ESI/MS/MS MRM system (Waters ACQUITY UPLC (Waters, Milford, MA, USA) and QTRAP 5500 (AB Sciex, Foster City, CA, USA), 25(OH)D_3_(6,19,19-D_3_) was used as the internal standard.

### Tumor xenografts

Subcutaneous xenografts formation was conducted as described previously^23^, 1 × 10^6^ pH 7.4 or pH 6.8 treated GSC2 cells were injected subcutaneously to the left flank of nude mice in 0.1 mL of PBS. After 6 days, 1 μM/kg NaHCO_3_ in 0.1 mL of sterile water was injected subcutaneously to the left flank of nude mice every other day. After 27 days the tumor volumes of each group (*n* = 5) were estimated using the formula *V* = *ab*^2^/2 (*V*, volumes; *a*, length; *b*, width).

For intracranial xenografts, BALB/c-nu mice received 1 × 10^5^ pH 7.4 or pH 6.8 treated GSC2 cells in 5 μL of culture medium by stereotactic injection to the right neostriatum. 1 μg/kg 1α,25(OH)_2_D_3_ was administered intraperitoneally 6 days a week from day 5. Groups of mice (*n* = 5) were undergoing a magnetic resonance imaging (State Key Laboratory of Bioactive Substance and Function of Natural Medicines, Institute of Materia Medica, Chinese Academy of Medical Sciences and Peking Union Medical College, Beijing) after 17 days. Animal protocol was approved by the Animal Ethics Committee of Peking Union medical college.

### Statistic analysis

Data were expressed as the means ± SDs and were analyzed by SPSS 14.0 software (SPSS, IL). Student’s *t*-test was used for statistical analysis, and a *P* < 0.05 was considered statistically significant.

### Study approval

Glioma samples were classified according to the Histological Grades of Tumors of the Nervous System (3th edition, WHO, 2000). Informed consent was obtained before surgery. The study was approved by the Medical Ethics Committee of Beijing Tiantan Hospital.

## Results

### Acidosis promotes cancer stem cell phenotypes and increases mitochondrial metabolism

**Fig. 1:**
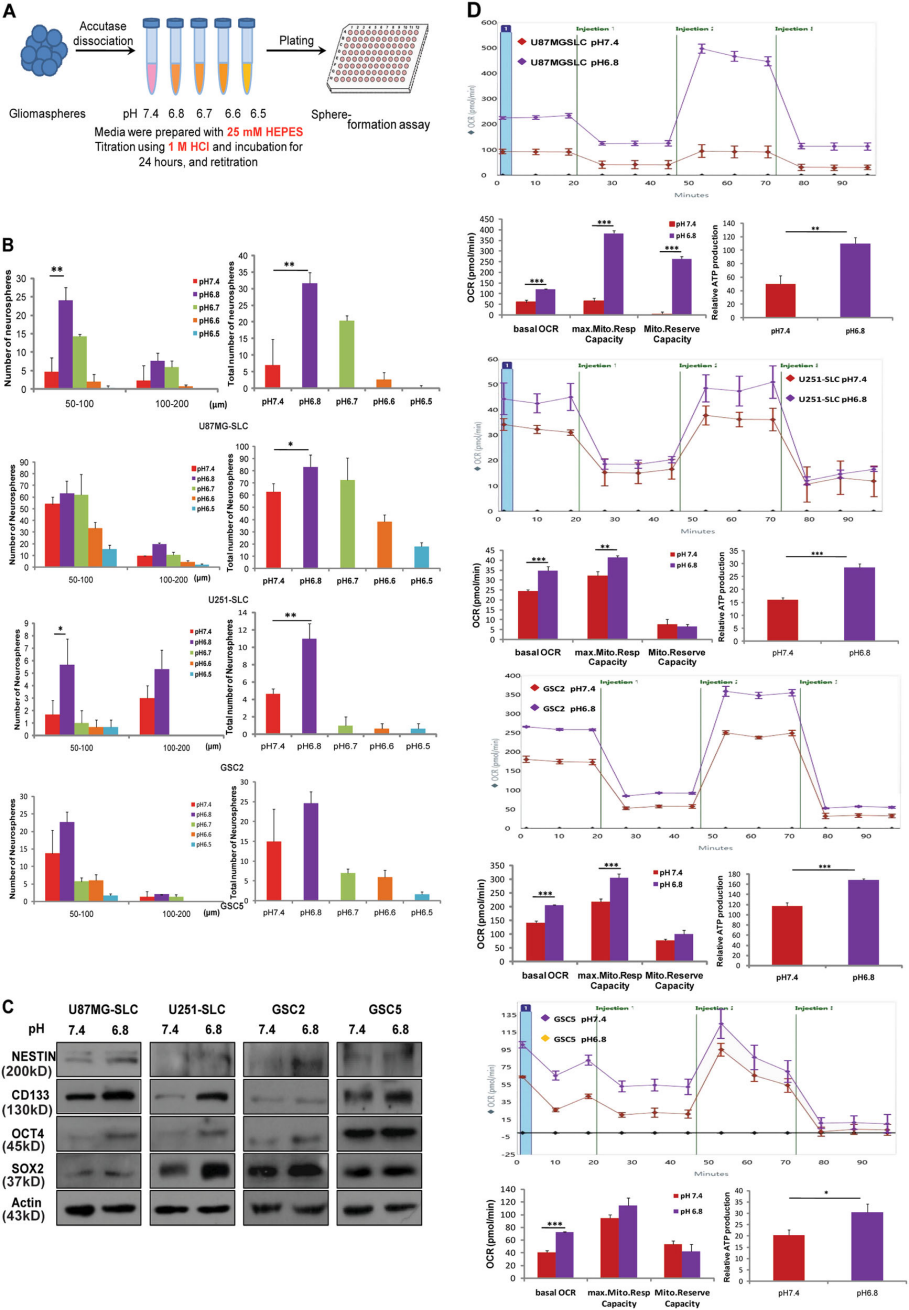
Acidic microenvironment drives self-renew and mitochondrial metabolism. **a** Schematic diagram of pH treatment of SLCs. Adding 25 mM HEPES buffer into the culture medium. The pH values of the medium were set as 7.4, 6.8, 6.7, 6.6, and 6.5. Using neurosphere formation assay to detect the effect of different pH values on the sphere formation ability of SLCs. **b** Neurosphere formation assay to determine the best pH value for the self-renewal of SLCs. The volume and number of neurospheres (diameters larger than 50 µm) of U87MG-SLC, U251-SLC, GSC2, and GSC5 under pH 7.4, 6.8, 6.7, 6.6, and 6.5 conditions (*P < 0.05; **P < 0.01, Student’s *t*-test). **c** Immunoblotting of the expression of stemness markers NESTIN, CD133, OCT4, and SOX2 in pH 7.4-treated and pH 6.8-treated U87MG-SLC, U251-SLC, GSC2, and GSC5 cells. **d** Respiration of mitochondria in SLCs/7.4 (red) and SLCs/6.8 (purple) U87MG-SLC, U251-SLC, GSC2, and GSC5 cells treated with oligomycin, FCCP, Antimycin A, and Rotenone. Oxygen consumption rate of basal respiration (basal OCR), maximal respiration (max. Mito. Resp Capacity), spare respiratory capacity (Mito.Reserve Capacity), and ATP production were shown (bottle panel; *P < 0.05; **P < 0.01; ***P < 0.001, Student’s *t*-test)

To determine the best pH value for the growth of SLCs, we cultured the SLCs (U87MG-SLCs, U251-SLCs, GSC2, and GSC5) which we previously established^21^ in mediums with five different pH values (7.4, 6.8, 6.7, 6.6, and 6.5), and their self-renewal abilities were examined (Fig. 1a). Under acidic pH 6.8, the volume and number of neurospheres of four SLCs were elevated compared to the other four pH values (Fig. 1b). This is consistent with previous report that the pH value in glioma tissues was 6.8 and can simulate the state of the acidic environment in vivo. Therefore, we chose pH 6.8 as the acidic treatment condition and pH 7.4 as the normal treatment condition to conduct the subsequent assay. To confirm the influence of acidosis on the stemness of SLCs, we detected the expression of four stemness markers NESTIN, CD133, OCT4, and SOX2 with western blot. The results showed that the stemness markers of acidic-treated SLCs were increased (Fig. 1c), which is consistent with the enhancement of neurosphere formation.

We further investigated the mitochondrial respiration of SLCs/7.4 and SLCs/6.8 to explore the effect of acidosis on mitochondrial metabolism, results showed that the basal respiration, maximal respiration, and ATP production were increased in acidic-treated SLCs (Fig. 1d). Moreover, we also measured the OCR of pH 7.4 and pH 6.8 treated U87MG, U251, GSC2, and GSC5 differentiated cells, and found there were no significant increase of basal respiration, maximal respiration or ATP production (Figure S1A). Taken together, these results demonstrated that acidosis promotes self-renewal ability, expression of stemness markers, and mitochondrial respiration of SLCs.

### Identification of five candidates through microarray analysis

To explore the underlying mechanism of the observation that the stemness and mitochondrial respiration were different under acidosis, the mRNA and long noncoding RNA (lncRNA) microarray analysis was used to identify the differences between pH 7.4 and pH 6.8 treated GSC5 cells. And we found that 888 genes and 1826 lncRNAs were upregulated while 70 genes and 83 lncRNAs were downregulated in pH 6.8 treated GSC5 (fold change > 2). Then we further selected seven genes from 958 genes (888 upregulated and 70 downregulated) and seven lncRNAs from 1909 lncRNAs (1826 upregulated and 83 downregulated) that exhibited 10-fold change greater than pH 7.4 treated GSC5 (Figures S2A and S2B). The increased mitochondrial respiration led us to select five genes from 958 genes that were related to mitochondrial function (Figure S2C).

**Fig. 2:**
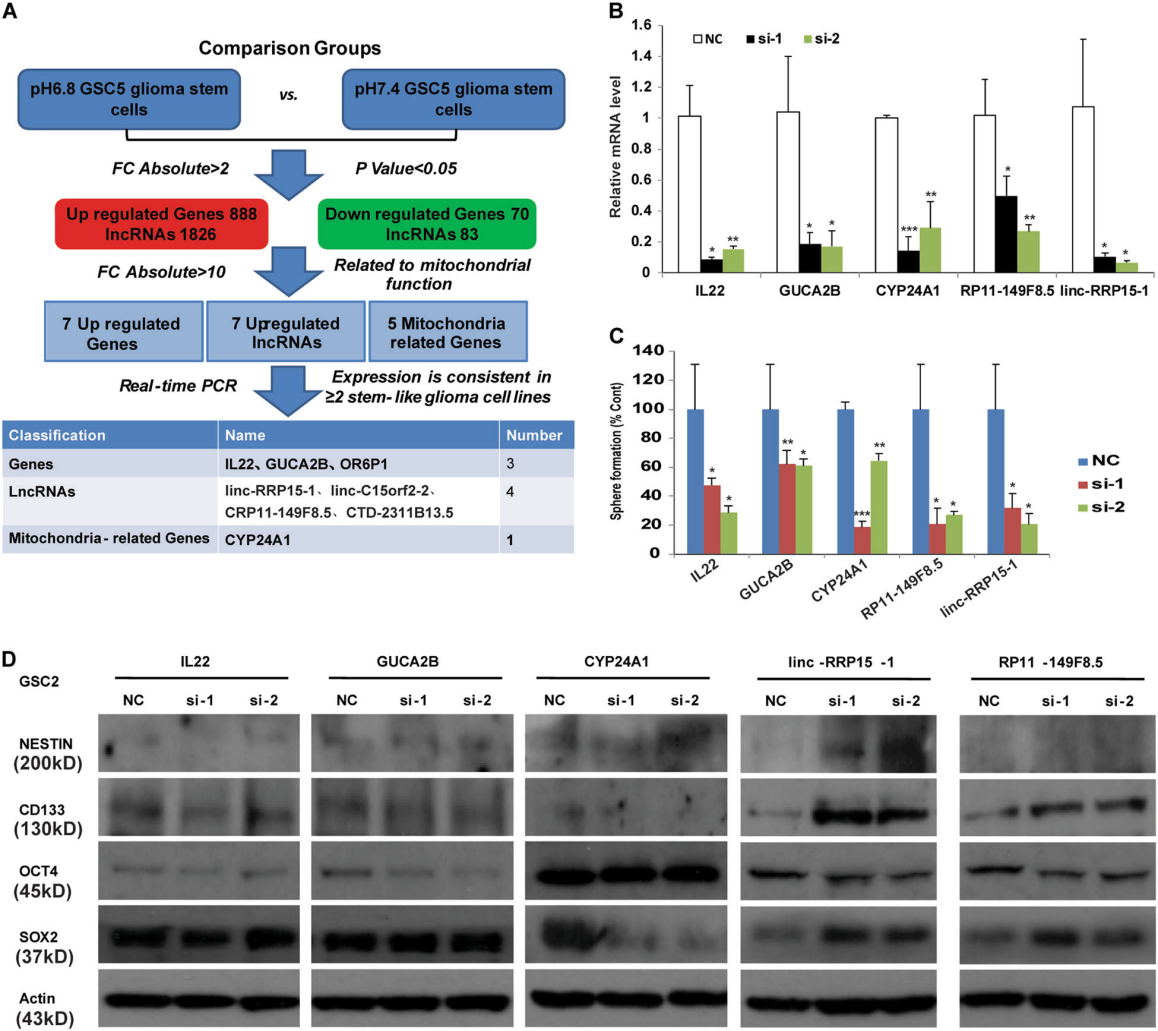
Screening three genes and two lncRNAs that were changed under acidosis. **a** Principle and process of screening. pH 7.4-treated and pH 6.8-treated GSC5 cells were used to conduct microarray analysis. Relative quantitative real-time PCR were used to verify the expression of mRNAs and lncRNAs. The selected eight genes and lncRNAs were shown (bottom panel). **b** Relative RNA levels of IL22, GUCA2B, CYP24A1, and lncRNA RP11-149F8.5, linc-RRP15-1 transfected with targeting siRNA in GSC2 cells (*P < 0.05; **P < 0.01; ***P < 0.001, Student’s *t*-test). **c** Neurosphere formation ability of SLCs when knockdown of IL22, GUCA2B, CYP24A1, and lncRNA RP11-149 f8. 5, linc-RRP15-1 using siRNA. Left: Neurosphere formation assay showed the number of neurospheres (diameters larger than 50 µm) formed from GSC2 cells (*P < 0.05; **P < 0.01; ***P < 0.001, Student’s *t*-test). Right: phase contrast photomicrographs showed morphological change of GSC2 cells after knockdown of GUCA2B (bar = 100 μm, left; bar = 50 μm, right). **d** Immunoblotting of the expression of stemness markers NESTIN, CD133, OCT4, and SOX2 in U251-SLC that transfected with targeting siRNA of IL22, GUCA2B, CYP24A1, and lncRNA RP11-149 f8. 5, linc-RRP15-1

Next, we used relative quantitative real-time PCR to examine the expression of selected genes and lncRNAs in U87MG-SLCs, U251-SLCs, GSC2, and GSC5 (Figures S2A, S2B, S2C, and S2D). According to the expression change (consistent in greater than two SLCs), three genes, four lncRNAs, and one mitochondrial function-related genes were obtained as candidate genes (Fig. 2a).

We further performed functional screening by knocking down the candidate genes IL22 (interleukin 22), GUCA2B (guanylate cyclase activator 2B), CYP24A1 (cytochrome P450, family 24, subfamily A, polypeptide 1), and lncRNAs (RP11-149F8.5 and linc-RRP15-1) (Fig. 2b). Neurosphere formation assay showed that silencing the five candidates significantly impaired the self-renewal of SLCs (Fig. 2c). We then used immunoblotting experiments to examine the expression of stemness markers in U251-SLCs, GSC2, and GSC5, and found that the expression of stemness markers decreased when silencing IL22, GUCA2B, CYP24A1. In contrast, no obvious change was observed when lncRNA RP11-149F8.5 and linc-RRP15-1 were knockdown (Figs. 2d, S3A and S3B). According to these data, we screened out five candidates (IL22, GUCA2B, CYP24A1 and lncRNA RP11-149F8.5, linc-RRP15-1) through microarray analysis, and found that knockdown of them impaired the self-renewal ability of SLCs.

### Influence of five candidates on cancer stem cell phenotypes and mitochondrial metabolism under acidosis

**Fig. 3:**
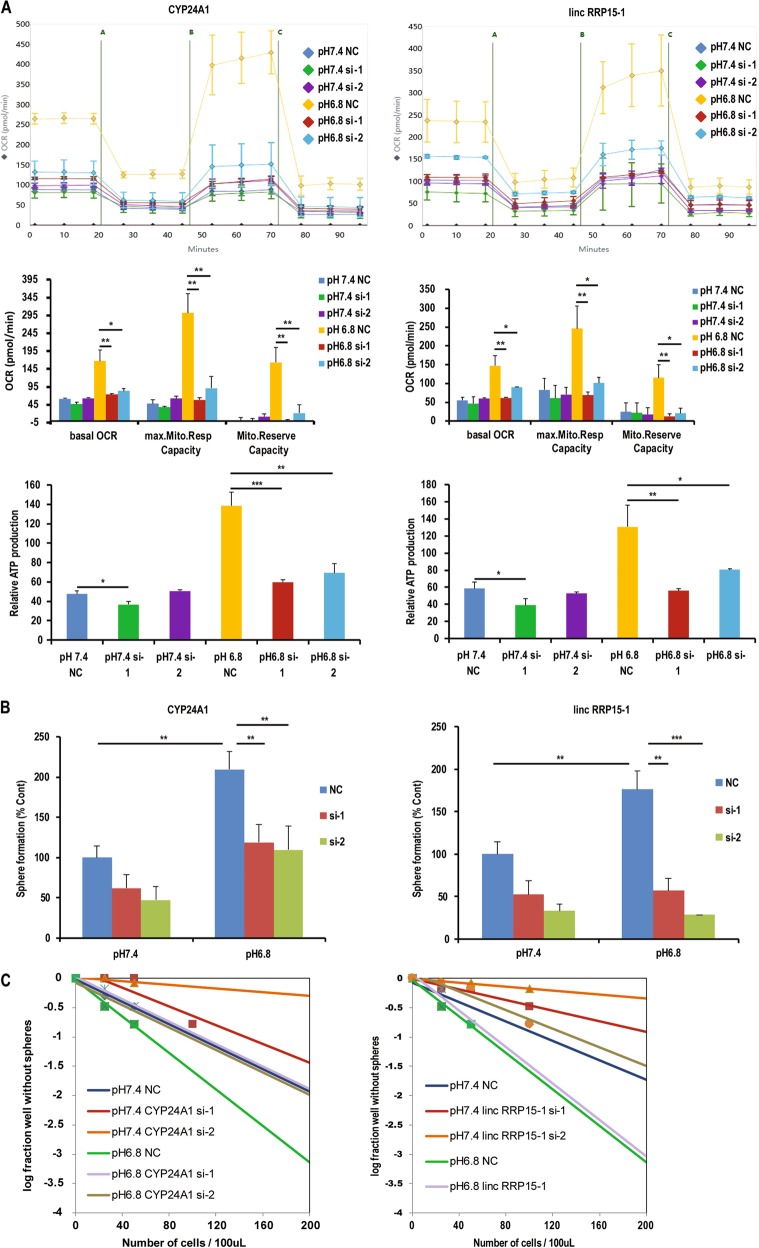
Analysis the influence of five candidates on the stemness and mitochondrial respiration of SLCs. **a** Respiration of mitochondria in U251-SLCs that knockdown of CYP24A1 and linc-RRP15-1 under pH 7.4 or pH 6.8 culture conditions and treated with oligomycin (named as “A”), FCCP (named as “B”), Antimycin A, and Rotenone (named as “C”). Oxygen consumption rate of basal respiration (basal OCR), maximal respiration (max. Mito. Resp Capacity), spare respiratory capacity (Mito.Reserve Capacity), and ATP production were shown. **b** Self-renewal ability of SLCs while knockdown of two candidates under pH 7.4 or pH 6.8 culture conditions. Neurosphere formation assay showed the number of neurospheres (diameters larger than 50 µm) formed from U251-SLCs that transfected with targeting siRNA of CYP24A1 and lncRNA linc-RRP15-1, *P < 0.05; **P < 0.01; ***P < 0.001, Student’s *t*-test. **c** Limiting dilution assay of pH 7.4-treated and pH 6.8-treated U251-SLCs that knockdown of CYP24A1 and lncRNA linc-RRP15-1. Cells were diluted into 200, 100, 50, 25, and 0 per 100 μL, wells not containing spheres (diameter that larger than 50 μm) for each cell plating density was calculated after 2 weeks

To further determine the effect of the five candidates on stemness and mitochondrial respiration in SLCs, we first used neurosphere formation assay to examine the self-renewal ability in U251-SLCs. Results showed that the increased number of neurospheres was blunted under acidosis by knockdown of IL22, GUCA2B, CYP24A1, while it was completely inhibited when silencing lncRNA RP11-149F8.5 and linc-RRP15-1 (Figs. 3b and S3C). Meanwhile, the results of limiting dilution assay confirmed that knockdown of the five candidates could partly reversed the increase of self-renewal ability under acidosis (Figs. 3c and S3D). Next, we aimed to explore the influence of silencing the five candidates on the mitochondrial respiration. Only knocking down CYP24A1 or linc-RRP15-1 could cause the decrease of mitochondrial respiration under acidosis (Figs. 3a and S3E). In summary, we confirmed that CYP24 A1 knockdown could rescue acidosis-induced self-renewal ability and mitochondrial metabolism in SLCs.

### The expression of CYP24A1 was relatively high in grade IV glioma tissues and acidic microenvironment

**Fig. 4:**
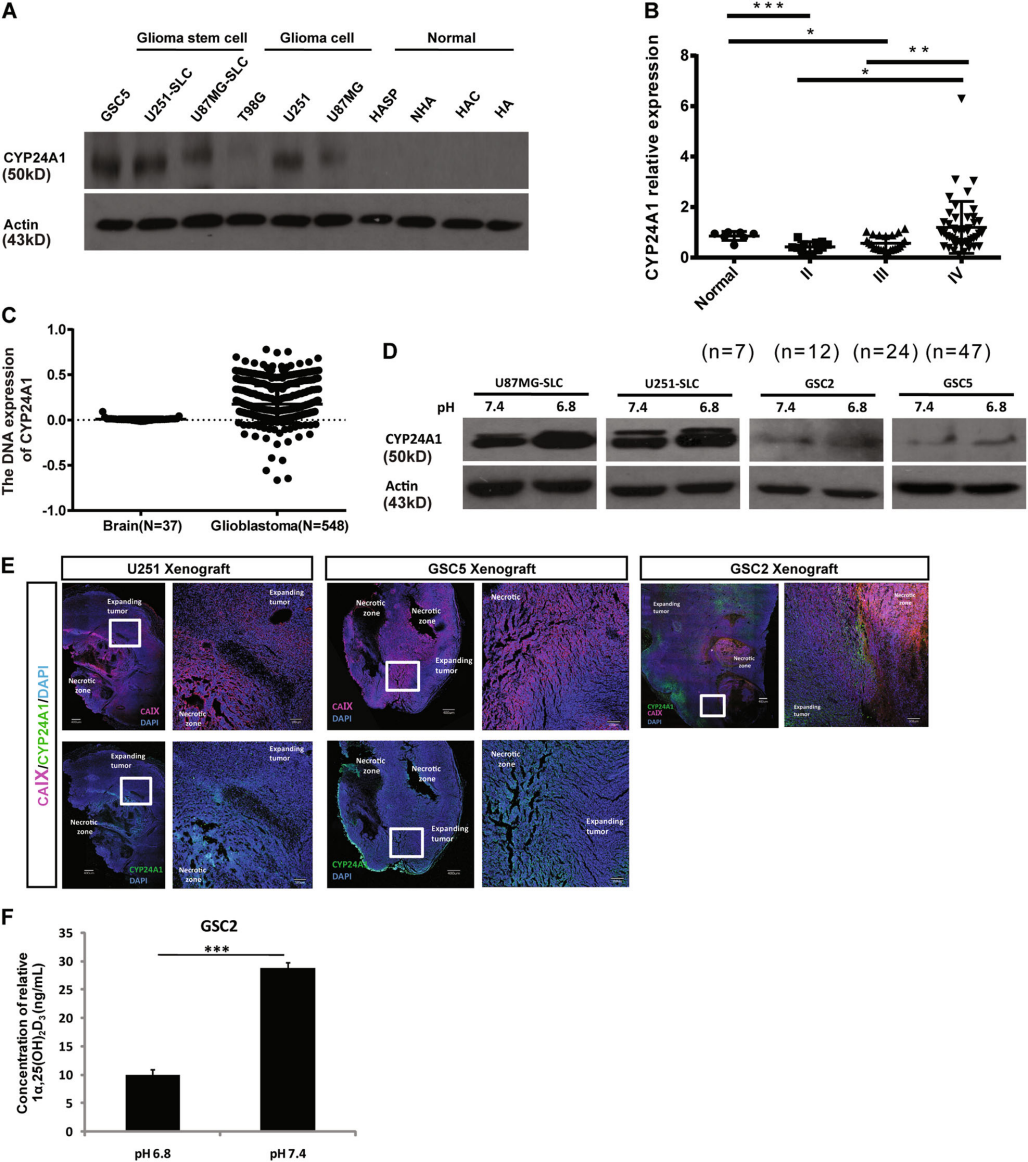
CYP24A1 was highly expressed in acidic microenvironment and high grade glioma tissues. **a** Immunoblotting of the expression of CYP24A1 in HA, HAC, NHA, and HASP normal human astrocytes, U87MG, U251, and T98G glioma cell lines and U87MG-SLC, U251-SLC, and GSC5 stem cell-like glioma cells. **b** The relative CYP24A1 protein levels in seven normal brain tissues and in 83 glioma tissues (12 grade II, 24 grade III, and 47 grade IV glioma tissues); actin was used as a control. The relative intensity values of CYP24A1/actin were analyzed by Image J software. **c** Immunoblotting of the expression of CYP24A1 in U87MG-SLC, U251-SLC, GSC2, and GSC5 cells under pH 7.4 or pH 6.8 conditions. **d** Immunofluorescence analysis of CYP24A1 (green) and carbonic anhydrase IX (CA IX, red) merged with nuclear DAPI staining (blue) in xenografts developed from U251, GSC2, and GSC5 cells (bar = 400 μm, left; bar = 100 μm, right). **e** Quantification of endogenous 1α,25(OH)_2_D_3_ in pH 7.4-treated and pH 6.8-treated GSC2 cells. The values shown are the means ± SD of at least three independent experiments (***P *<* 0.001, Student’s *t*-test)

CYP24A1 is a key enzyme that is involved in the catabolism of vitamine D. It can catalyze 25-OH-D_3_ and 1α,25-(OH)_2_D_3_ into 24-hydroxylated metabolites^26^.To evaluate the correlation between the expression of CYP24A1 and the malignancy of glioma, and the expression pattern of CYP24A1 in acidic microenvironment in vivo is also important. We first analyzed the expression of CYP24A1 in pH 7.4 and pH 6.8 treated U87MG-SLCs, U251-SLCs, GSC2, and GSC5 cells, as well as four normal cell lines, three glioma cell lines, and four SLCs. CYP24A1 was overexpressed in pH 6.8-treated SLCs and showed the highest level in SLCs compared to other cell lines (Fig. 4a–c). Results from seven normal brain tissues and 83 glioma tissues showed that CYP24A1 protein level in grade IV glioma tissues was higher than that in grade II and grade III glioma tissues (Figs. 4b and S6A).

Next, we injected U251, GSC2, and GSC5 cells subcutaneously in nude mice to obtain glioma xenografts, using carbonic anhydrase IX as an acidic indicator, we examined the expression pattern of CYP24A1 in vivo. The hypoxic and acidic microenvironment existed around the necrotic zone and the oxygen and pH were relatively high in tumor expanding area. We found that carbonic anhydrase IX was highly expressed around necrotic zone, which indicated high hydrogen ion concentration in this area, and the expression of CYP24A1 was similar with the expression of carbonic anhydrase IX (Figs. 4d and S6B). This confirms the relatively high expression of CYP24A1 in acidic microenvironment. In order to determine whether the acidic microenvironment affects 1α,25-(OH)_2_D_3_ by regulating CYP24A1 expression, we used HPLC–MS to analyze the production of 1α,25-(OH)_2_D_3_ in pH 7.4 and pH 6.8 treated GSC2 cells. The quantity of 1α,25-(OH)_2_D_3_ decreased significantly in GSC2 cells under acidosis (Fig. 4e). All of these results demonstrated that CYP24A1 was highly expressed in malignant glioma tissues and acidic microenvironment, where the CSCs were existed. This accelerated the catabolism of 1α,25-(OH)_2_D_3_, resulting in reduced 1α,25-(OH)_2_D_3_ in acidic microenvironment. Therefore, we proved that CYP24A1 may be positively related to the malignancy of glioma and the stemness of GSCs.

### 1α,25(OH)_2_D_3_ impaired the stemness and restrained the mitochondrial activity of SLCs in acidic microenvironment

**Fig. 5:**
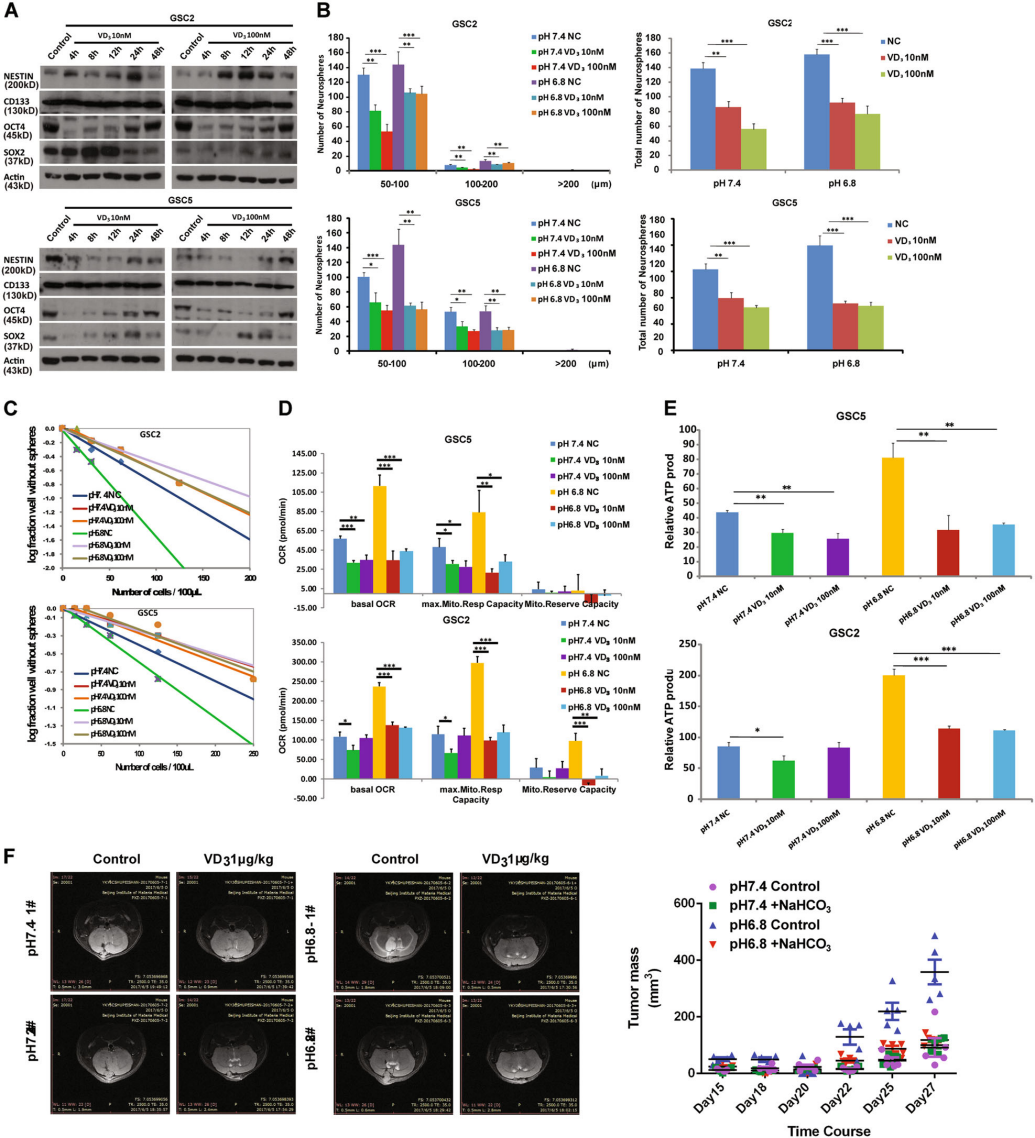
1α,25(OH)2D3 inhibited the stemness and impaired the mitochondrial respiration and ATP production in acidic condition. **a** Immunoblotting of the expression of stemness markers NESTIN, CD133, OCT4, and SOX2 in GSC2 and GSC5 cells that were treated with 1α,25(OH)_2_D_3_ 10 or 100 nM for 4, 8, 12, 24, and 48 h. **b** and **c** Self-renewal ability of SLCs with 1α,25(OH)_2_D_3_ treatment under pH 7.4 or pH 6.8 culture conditions. Neurosphere formation assay showed the number of neurospheres (diameters larger than 50 µm) formed from GSC2 and GSC5 cells that were treated with 1α,25(OH)_2_D_3_ 10 or 100 nM (**b**), *P < 0.05; **P < 0.01; ***P < 0.001, Student’s *t*-test. Limiting dilution assay of pH 7.4-treated and pH 6.8-treated GSC2 and GSC5 cells were diluted into 250, 125, 62.5, 31.25, 15.625, and 0 per 100 μL that were treated with 1α,25(OH)_2_D_3_ 10 or 100 nM. Wells not containing spheres (diameter that are larger than 50 μm) for each cell plating density was calculated after 2 weeks (**c**). **d** and **e** Respiration of mitochondria in GSC2 and GSC5 cells that were treated with 1α,25(OH)_2_D_3_ 10 or 100 nM for 4 h under pH 7.4 or pH 6.8 culture conditions. Oxygen consumption rate of basal respiration (basal OCR), maximal respiration (max. Mito. Resp Capacity), spare respiratory capacity (Mito. Reserve Capacity), and ATP production were shown. *P < 0.05; **P < 0.01; ***P < 0.001, Student’s *t*-test. **f** The photos of magnetic resonance imaging of pH 7.4-treated or pH 6.8-treated GSC2 xenografts in nude mice treated intraperitoneally 6 days a week from day 5 with 1 μg/kg 1α,25(OH)_2_D_3_, Sesame oil was used as control (*n* = 5). **g** Tumor growth of pH 7.4 or pH 6.8-treated GSC2 xenografts. Sterile water was used as control to treat pH 7.4 (purple) or pH 6.8 (blue)-treated GSC2 xenografts, NaHCO_3_ was subcutaneously injected in pH 7.4 (green)-treated or pH 6.8 (red)-treated GSC2 xenografts (*n* = 5). pH 7.4 control vs. pH 6.8 control, *P* = 0.0012; pH 6.8 control vs. pH 6.8 + NaHCO_3_, *P* = 0.0006; Student’s *t*-test

1α,25(OH)_2_D_3_ has a variety of anticancer functions, it can inhibit the proliferation, invasion and metastasis, as well as induce the apoptosis and differentiation of cancer cells. Moreover, some studies have shown that 1α,25(OH)_2_D_3_ could target CSCs in prostate and breast tumor^27^. These drove us to investigate the function of 1α,25(OH)_2_D_3_ on SLCs. To confirm the effective concentration of 1α,25(OH)_2_D_3_ treatment, we used 10 and 100 nM to treat SLCs for 4, 8, 12, 24, and 48 h. The expression of stemness markers showed that NESTIN, OCT4, and SOX2 were decreased in GSC5 cells when treated with 1α,25(OH)_2_D_3_ 10 or 100 nM for 4–24 h, while OCT4 and SOX2 were decreased in GSC2 cells with 1α,25(OH)_2_D_3_ 100 nM treatment for 4–24 h, only OCT4 decreased with 1α,25(OH)_2_D_3_ 10 nM treatment, and NESTIN was increased in GSC2 cells (Fig. 5a). Meanwhile, OCT4 was decreased in U251-SLC when treated with 10 or 100 nM 1α,25(OH)_2_D_3_ (Figure S4A). In summary, 1α,25(OH)_2_D_3_ 10 or 100 nM treatment for 4–24 h could partly inhibit the expression of stemness markers especially OCT4 in SLCs, while treated for 48 h the effects were not significant.

Furthermore, we tested the effect of 1α,25(OH)_2_D_3_ on self-renewal ability. The size and number of neurospheres were reduced with 10 or 100 nM 1α,25-(OH)_2_D_3_ under pH 7.4 and pH 6.8 conditions, and the promotion of neurosphere formation was almost eliminated in GSC2, GSC5, and U251-SLCs under acidic (Figs. 5b, c and S4B). Taken together, these results indicated that 1α,25-(OH)_2_D_3_ could suppress the stemness of SLCs to some extent. Acidic microenvironment could upregulate the expression of CYP24A1 and lower the active 1α,25-(OH)_2_D_3_ to weaken the effect of 1α,25-(OH)_2_D_3_ on SLCs. It might be the pathway for SLCs to maintain their cancer stem cell phenotypes.

As 1α,25-(OH)_2_D_3_ repressed the stemness and self-renewal of SLCs, it is interesting whether 1α,25(OH)_2_D_3_ could affect the mitochondrial metabolism of SLCs. Then, we measured the mitochondrial oxygen consumption rate of SLCs pre-treated with 1α,25(OH)_2_D_3_ 10 or 100 nM for 8 h under pH 7.4 and pH 6.8 conditions. The basal respiration and maximal respiration of mitochondrial in GSC2 and GSC5 cells were significantly reduced with 1α,25(OH)_2_D_3_ treatment, and the increase of mitochondrial respiration was almost completely suppressed under pH 6.8 conditions (Figs. 5d and S5A). 1α,25(OH)_2_D_3_ inhibited the ATP production of mitochondria and rescued the acidosis triggered ATP enrichment (Fig. 5e).

Finally, to validate the effect of 1α,25(OH)_2_D_3_ on SLCs in vivo, we used pH 7.4 and pH 6.8 treated GSC2 cells to form xenografts in the brain of nude mice, then treated with 1α,25(OH)_2_D_3_ and evaluated tumor size. We found that treatment of 1 μg/kg 1α,25(OH)_2_D_3_ reduced the size of tumors derived from GSC2 cells, while the suppression of 1α,25(OH)_2_D_3_ was relatively obvious in tumors arising from pH 6.8-treated GSC2 cells (Fig. 5f). The results indicated that 1α,25(OH)_2_D_3_ restrained the tumorigenesis of acidic pH-treated SLCs and suggested that 1α,25(OH)_2_D_3_ may be a safe and effective compound to treat malignant glioma.

In addition, recent evidence showed that bicarbonate had a pivotal role in cancer therapy via change in pH value in tumor environment^28^. Therefore, we further investigated the effect of bicarbonate on the tumorigenic capacity of SLCs. pH 7.4-treated and pH 6.8-treated GSC2 cells were used to form subcutaneous xenografts. Then, we used NaHCO_3_ to treat the tumor tissue and found that the tumor mass arising from pH 6.8-treated GSC2 was significantly decreased (Fig. 5g). Thus our results confirmed that bicarbonate could inhibit the growth of SLCs in acidic microenvironment.

## Discussion

**Fig. 6:**
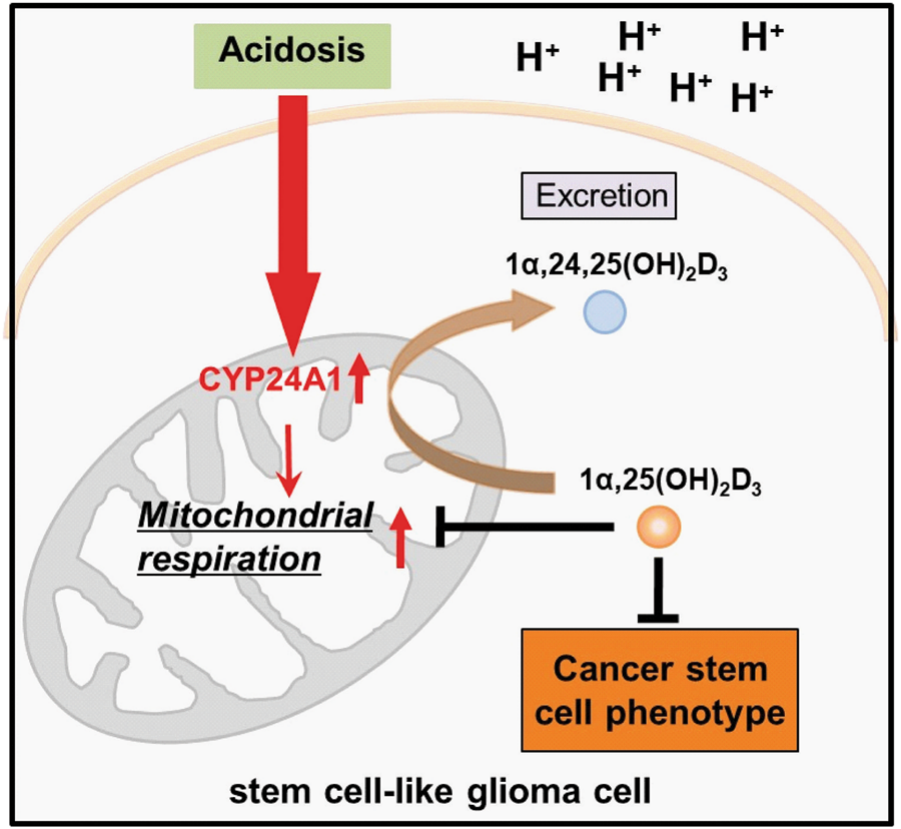
Schematic illustration of the regulation of acidosis-CYP24A1-1α,25(OH)2D3 axis in cancer stem cell phenotype of stem cell-like glioma cells. Extracellular acidic upregulated the expression of CYP24A1 and increased the activity of mitochondrial respiration. Meanwhile, CYP24A1 over-expression leads to the degradation of 1α,25(OH)_2_D_3_. While 1α,25(OH)_2_D_3_ could inhibit the cancer stem cell phenotype of stem cell-like glioma cells as well as impair the mitochondrial respiration

The results of our study showed that acidic microenvironment could promote and maintain the stemness of SLCs in malignant gliomas. Meanwhile, the activity of mitochondria and ATP production were increased to provide energy and biomacromolecule for the CSCs. The changes of stemness and mitochondrial dynamics were attributed to the upregulation of CYP24A1, a mitochondrial enzyme that degraded the active form of vitamin D^26^. Interestingly, the data indicated that the active form of vitamin D (1α,25(OH)_2_D_3_, also named calcitriol) could inhibit the stemness of SLCs (Fig. 5d). Our study underlined that the CYP24A1–vitamin D axis may be a key determinant of SLCs survival in acidic microenvironment and pointed out the potential value for the use of vitamin D to target CSCs (Fig. 6).

Acidic microenvironment could regulate the growth of malignant glioma cells and their sensitivity to chemotherapy. As hypoxic tissues become more acidic, tumor cells will go into a dormant state, escaping chemotherapy and immunotherapy. The usage of NaHCO_3_ could effectively reverse the acidic environment in cancer tissue and made dormant cancer cells sensitive to current therapies^29^. Under the condition of pH 6.6, the growth of all glioma cells were inhibited, but their sensitivity to radiotherapy was verified. In addition, pH 6.6 conditions could increase the cytotoxicity effect of lomustine drugs, but protect the cells from the cytotoxic effect of topotecan, vincristine, teniposide, and cisplatin^22^, providing a reference for the personal radiation and chemotherapy treatment. Our studies proved that pH 6.8 acidic conditions increased neurosphere formation ability and tumorigenic capacity of SLCs, implying that acidic microenvironment promoted the transformation of glioma cells into malignant cells, such as GSCs, and made us to explore the anti-chemotherapy and anti-radiotherapy ability of GSCs while normalizing tumor acidic microenvironment.

Although the generation of acidic microenvironment partly is due to the metabolic phenotypes of cancer cells^30,31^, evidence showed that acidosis may lead to metabolic reprogramming of cancer cells^32^. Here, we found that acidic condition could affect mitochondrial activity and upregulate the expression of CYP24A1 which expressed in the inner membrane of mitochondria, by which to satisfy the biological energy and biosynthesis requirements of CSCs through using TCA cycle and mitochondrial function. The classical methods of isolation of glioma stem cells (GSCs) are side population analysis, CD133-labeled cell sorting and neurosphere growth^33,34^. However, these methods only enriched a fraction of GSCs, and the isolated cells are still heterogeneous population. The results that acidosis promoted the cancer stem cell properties of SLCs remind us of acidic condition may be a method to further isolate and purify GSCs.

Vitamin D has been thought to play a potential role in cancer therapy. Research showed that the active metabolite of vitamin D (1α, 25(OH)_2_D_3_) could restrain tumor growth mainly through the vitamin D response elements of target genes^35–38^. This is correlated with the inhibition of proliferation and angiogenesis, induction of differentiation and apoptosis function of 1α, 25(OH)_2_D_3_ in cancer^27,39^. Epidemiological studies implied that vitamin D deficiency was related to the increase of multiple cancer incidence in the world^40^. Evidence showed that in the area of less sunshine the incidence of colon and prostate cancer was increased^41,42^. Moreover, 1α,25(OH)_2_D_3_ lead to senescence, cell-cycle arrest, and differentiation of prostate stem cells^43^. A vitamin D derivative could inhibit the tumor growth and the expression of cancer stem cell marker CD44 in human breast cancer^44^. Additionally, we found that calcitriol repressed the self-renewal and cancer stem cell marker expression in SLCs, these results revealed the function of vitamin D on the suppression of GSCs in malignant glioma.

CYP24A1 is a rate-limiting enzyme for the catabolism of 1α,25(OH)_2_D_3_^45^. It has been considered to be a potential oncogene in breast cancer^46^. The overexpression of it in lung adenocarcinoma was associated with patients’ poor survival because of the rapid clearance of 1α,25(OH)_2_D_3_ and the abrogation of antiproliferative effects^47^. The combination use of CYP24A1 inhibitor and calcitriol exhibited a more effective anticancer function in prostate cancer^48,49^. We found a relatively high expression of CYP24A1 in high level glioma tissue (Figs. 4b and S6A). To further explore the role of the vitamin D metabolic pathway in glioma, we examined the expression of vitamin D receptors and CYP27A1 and CYP27B1, key enzymes involved in synthesis of 25(OH)D_3_ and 1α,25(OH)_2_D_3_ in glioma tissues. The expression of CYP27A1 is relatively low in high level glioma tissue, the expression of CYP27B1 is elevated in glioma tissues and increased significantly in grade IV glioma tissue (Figures S7A and S7B).

Here, we discovered that CYP24A1 was highly expressed in SLCs under acidosis. Recent studies have reported that Smad5 can feel the changes in intracellular pH value, and shuttles between nuclear and cytoplasm. The increase in intracellular pH can make protons dissociate from the MH1 domain of Smad5, resulting in its export to cytoplasm and interaction with HK1, thus accelerates glycolysis^50^. So we inquired whether there was a related charged structure in CYP24A1 protein, and found that CYP24A1 had a P450 domain^51^, but whether this structure could detect proton concentration is unknown. Because of the elevated expression of CYP24A1 mRNA in acidic environments, we examined the effect of acidic environment on the CYP24A1 promoter region in SLCs and found no significant changes (data not shown). Whether there is another transcriptional or post-transcriptional regulatory mechanism requires further study. The over expression of CYP24A1 resulted in the reduction of 1α,25(OH)_2_D_3_, thus reversed 1α,25(OH)_2_D_3_ caused stemness decrease in SLCs and may increase the malignancy, as well as therapy resistence of SLCs. This study suggested that CYP24A1 may play an important role in malignant glioma and provided a new strategy to use CYP24A1 inhibitor and calcitriol for glioma therapy.

## Supplementary information

supplemental material

## References

[1] Albertson DG (2000). Quantitative mapping of amplicon structure by array CGH identifies CYP24 as a candidate oncogene. Nat. Genet..

[2] Amo T (2008). Experimental assessment of bioenergetic differences caused by the common European mitochondrial DNA haplogroups H and T. Gene.

[3] Cairns RA, Harris IS, Mak TW (2011). Regulation of cancer cell metabolism. Nat. Rev. Cancer.

[4] Chao, M. et al. A nonrandomized cohort and a randomized study of local control of large hepatocarcinoma by targeting intratumoral lactic acidosis. *eLife* **5**; e15691 (2016). 10.7554/eLife.15691 PMC4970867 27481188

[5] Chen G (2011). CYP24A1 is an independent prognostic marker of survival in patients with lung adenocarcinoma. Clin. Cancer Res..

[6] Chen H (2003). Heterogeneous nuclear ribonucleoprotein (hnRNP) binding to hormone response elements: a cause of vitamin D resistance. Proc. Natl Acad. Sci. USA.

[7] Chen H (2000). (2000) The vitamin D response element-binding protein. A novel dominant-negative regulator of vitamin D-directed transactivation. J. Biol. Chem..

[8] Corbet C (2014). The SIRT1/HIF2alpha axis drives reductive glutamine metabolism under chronic acidosis and alters tumor response to therapy. Cancer Res..

[9] Corbet C, Feron O (2017). Tumour acidosis: from the passenger to the driver’s seat. Nat. Rev. Cancer.

[10] Corbet C (2016). Acidosis drives the reprogramming of fatty acid metabolism in cancer cells through changes in mitochondrial and histone acetylation. Cell. Metab..

[11] Deeb KK, Trump DL, Johnson CS (2007). Vitamin D signalling pathways in cancer: potential for anticancer therapeutics. Nat. Rev. Cancer.

[12] Fang Y (2017). Smad5 acts as an intracellular pH messenger and maintains bioenergetic homeostasis. Cell Res..

[13] Feldman D (2014). The role of vitamin D in reducing cancer risk and progression. Nat. Rev. Cancer.

[14] Filatova A (2016). Acidosis acts through HSP90 in a PHD/VHL-independent manner to promote HIF function and stem cell maintenance in glioma. Cancer Res..

[15] Fretz JA (2006). 1,25-Dihydroxyvitamin D3 regulates the expression of low-density lipoprotein receptor-related protein 5 via deoxyribonucleic acid sequence elements located downstream of the start site of transcription. Mol. Endocrinol..

[16] Fukumura D (2001). Hypoxia and acidosis independently up-regulate vascular endothelial growth factor transcription in brain tumors in vivo. Cancer Res..

[17] Garland CF, Garland FC (1980). Do sunlight and vitamin D reduce the likelihood of colon cancer?. Int. J. Epidemiol..

[18] Gatenby RA, Gillies RJ (2004). Why do cancers have high aerobic glycolysis?. Nat. Rev. Cancer.

[19] Gerweck LE, Seetharaman K (1996). Cellular pH gradient in tumor versus normal tissue: potential exploitation for the treatment of cancer. Cancer Res..

[20] Grant WB (2012). Ecological studies of the UVB-vitamin D-cancer hypothesis. Anticancer Res..

[21] Hanchette CL, Schwartz GG (1992). Geographic patterns of prostate cancer mortality. Evidence for a protective effect of ultraviolet radiation. Cancer.

[22] Jacobs ET (2013). CYP24A1 and CYP27B1 polymorphisms modulate vitamin D metabolism in colon cancer cells. Cancer Res..

[23] Xie Q (2015). Mitochondrial control by DRP1 in brain tumor initiating cells. Nat. Neurosci..

[24] Xu L, Fukumura D, Jain RK (2002). Acidic extracellular pH induces vascular endothelial growth factor (VEGF) in human glioblastoma cells via ERK1/2 MAPK signaling pathway: mechanism of low pH-induced VEGF. J. Biol. Chem..

[25] Zhao M (2016). GSH-dependent antioxidant defense contributes to the acclimation of colon cancer cells to acidic microenvironment. Cell Cycle.

[26] Haussler MR (1998). The nuclear vitamin D receptor: biological and molecular regulatory properties revealed. J. Bone Mineral Res..

[27] Hjelmeland AB (2011). Acidic stress promotes a glioma stem cell phenotype. Cell Death Differ..

[28] Hu PS (2017). NSPc1 promotes cancer stem cell self-renewal by repressing the synthesis of all-trans retinoic acid via targeting RDH16 in malignant glioma. Oncogene.

[29] Walton ZE (2018). Acid suspends the circadian clock in hypoxia through inhibition of mTOR. Cell.

[30] Jain RK (2013). Normalizing tumor microenvironment to treat cancer: bench to bedside to biomarkers. J. Clin. Oncol..

[31] Jiang T (2016). CGCG clinical practice guidelines for the management of adult diffuse gliomas. Cancer Lett..

[32] Jones G, Prosser DE, Kaufmann M (2012). 25-Hydroxyvitamin D-24-hydroxylase (CYP24A1): its important role in the degradation of vitamin D. Arch. Biochem. Biophys..

[33] Jones G, Prosser DE, Kaufmann M (2014). Cytochrome P450-mediated metabolism of vitamin D. J. Lipid Res..

[34] Kreso A, Dick JE (2014). Evolution of the cancer stem cell model. Cell Stem Cell.

[35] Lin X (2016). Interplay between PCBP2 and miRNA modulates ARHGDIA expression and function in glioma migration and invasion. Oncotarget.

[36] Longo DL (2016). In vivo imaging of tumor metabolism and acidosis by combining PET and MRI-CEST pH imaging. Cancer Res..

[37] Ly LH (1999). Liarozole acts synergistically with 1alpha,25-dihydroxyvitamin D3 to inhibit growth of DU 145 human prostate cancer cells by blocking 24-hydroxylase activity. Endocrinology.

[38] Maund SL (2011). Interleukin-1alpha mediates the antiproliferative effects of 1,25-dihydroxyvitamin D3 in prostate progenitor/stem cells. Cancer Res..

[39] Neri D, Supuran CT (2011). Interfering with pH regulation in tumours as a therapeutic strategy. Nat. Rev. Drug Discov..

[40] O’Brien CA, Kreso A, Jamieson CH (2010). Cancer stem cells and self-renewal. Clin. Cancer Res..

[41] Parks SK, Chiche J, Pouyssegur J (2013). Disrupting proton dynamics and energy metabolism for cancer therapy. Nat. Rev. Cancer.

[42] Reichert M (2002). Modulation of growth and radiochemosensitivity of human malignant glioma cells by acidosis. Cancer.

[43] Rochel N (2011). Common architecture of nuclear receptor heterodimers on DNA direct repeat elements with different spacings. Nat. Struct. Mol. Biol..

[44] Schonberg DL, Bao S, Rich JN (2013). Genomics informs glioblastoma biology. Nat. Genet..

[45] So JY (2011). A novel Gemini vitamin D analog represses the expression of a stem cell marker CD44 in breast cancer. Mol. Pharmacol..

[46] Swami S (2005). Genistein potentiates the growth inhibitory effects of 1,25-dihydroxyvitamin D3 in DU145 human prostate cancer cells: role of the direct inhibition of CYP24 enzyme activity. Mol. Cell. Endocrinol..

[47] Vlashi E (2011). Metabolic state of glioma stem cells and nontumorigenic cells. Proc. Natl Acad. Sci. USA.

[48] Wan F (2010). The utility and limitations of neurosphere assay, CD133 immunophenotyping and side population assay in glioma stem cell research. Brain Pathol..

[49] Wang J (2008). CD133 negative glioma cells form tumors in nude rats and give rise to CD133 positive cells. Int. J. Cancer.

[50] Williams AC, Collard TJ, Paraskeva C (1999). An acidic environment leads to p53 dependent induction of apoptosis in human adenoma and carcinoma cell lines: implications for clonal selection during colorectal carcinogenesis. Oncogene.

[51] Wu F (2016). RhoGDIalpha suppresses self-renewal and tumorigenesis of glioma stem cells. Oncotarget.

