# Supplementary material for: Acidosis enhances the self-renewal and mitochondrial respiration of stem cell-like glioma cells through CYP24A1-mediated reduction of vitamin D
Source: Cell Death Dis. 2019 Jan 10;10(1):25. doi: 10.1038/s41419-018-1242-1 (PMC6328565; doi:10.1038/s41419-018-1242-1)
Supplement: Supplementary file 1 — supplemental material [file 41419_2018_1242_MOESM1_ESM.docx]

**Supplementary Data**

**Fig. S1 Acidic microenvironment can not drives the mitochondrial respiration in glioma cells or differentiated SLCs.**


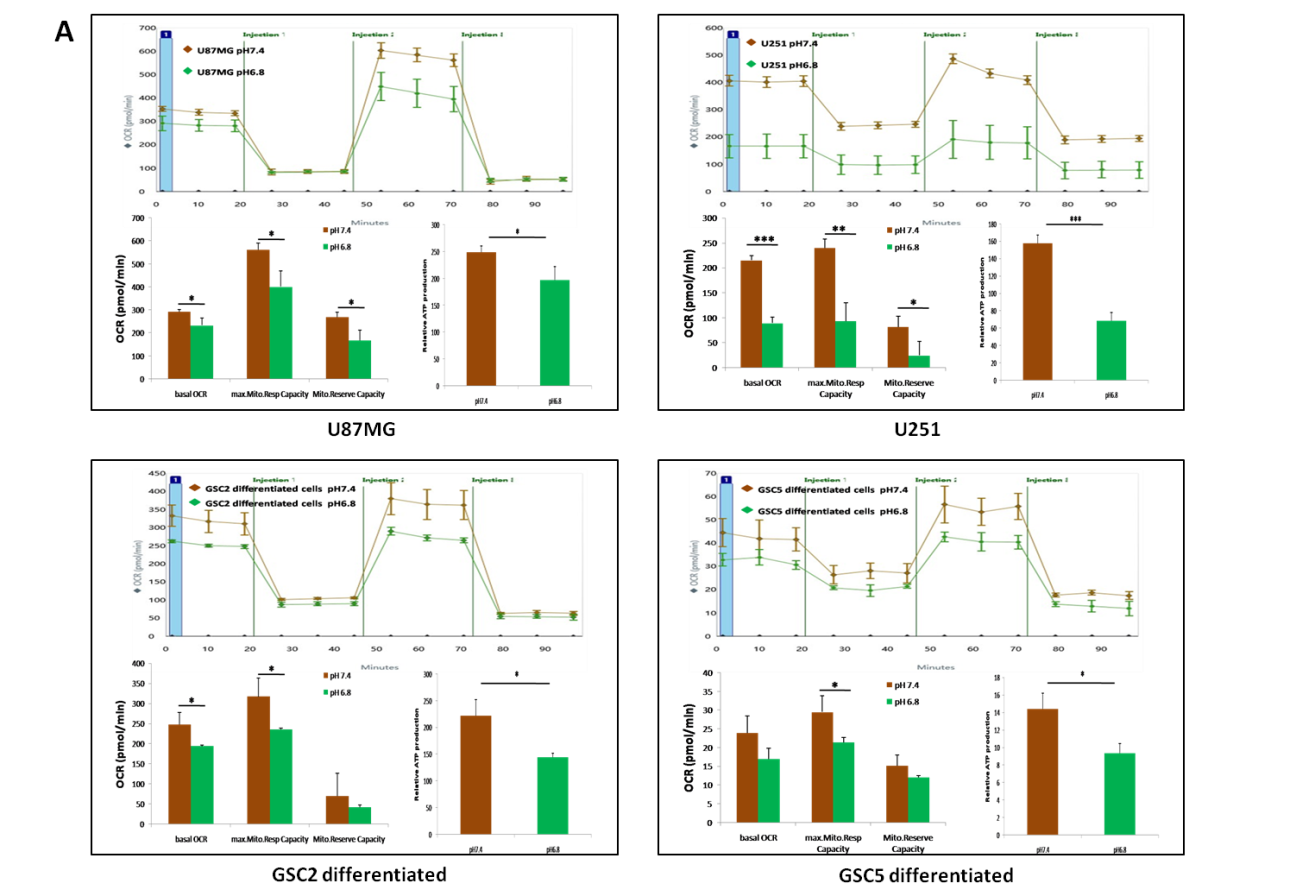


A．Respiration of mitochondria in U87MG, U251, GSC2 differentiated and GSC5 differentiated cells treated with oligomycin, FCCP, Antimycin A and Rotenone under pH7.4 (brown) and pH6.8 (green) conditions. Oxygen consumption rate of basal respiration (basal OCR), maximal respiration (max. Mito. Resp Capacity), spare respiratory capacity (Mito.Reserve Capacity) and ATP production were shown (bottom panel; * *P* < 0.05, ** *P* < 0.01, *** *P* < 0.001, Student’s t-test).

**Fig.S2** **Using relative quantitative real-time PCR to validate the microarray results.**


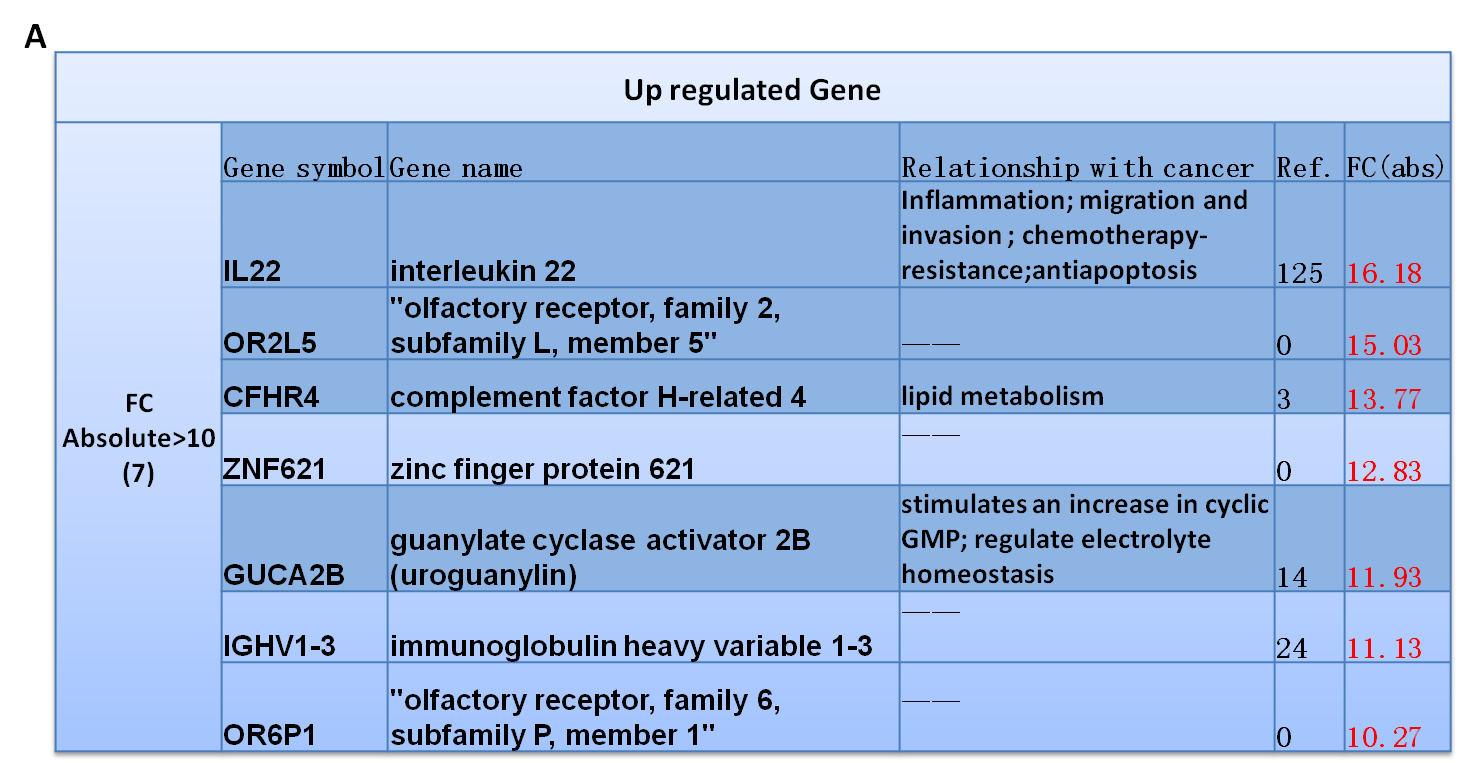


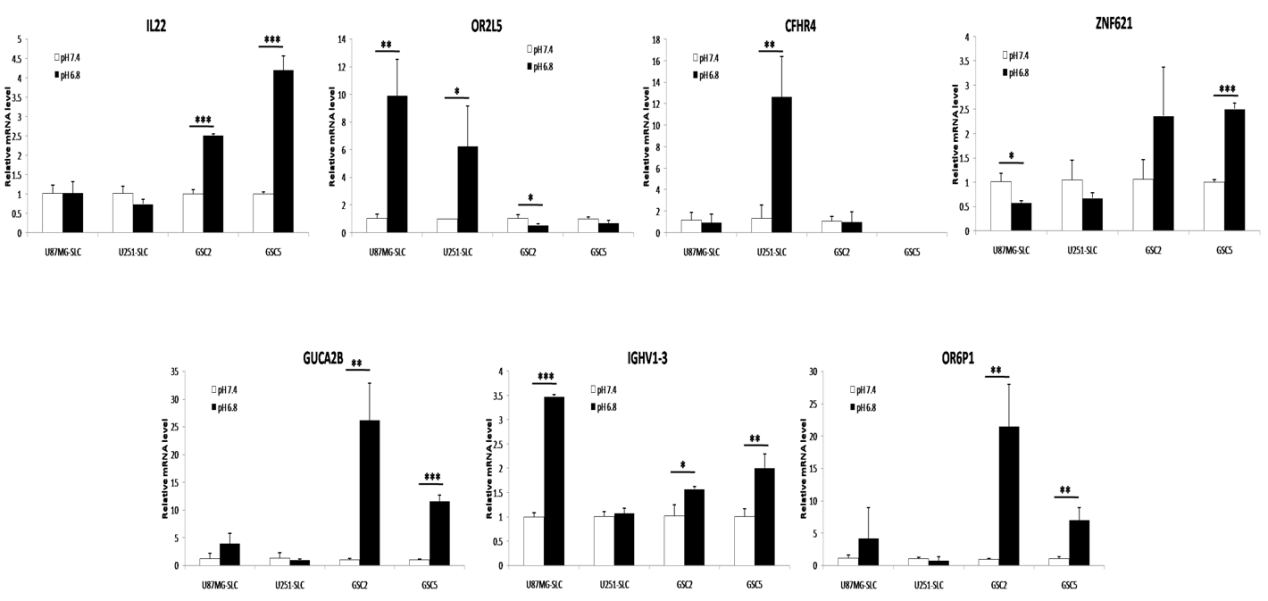


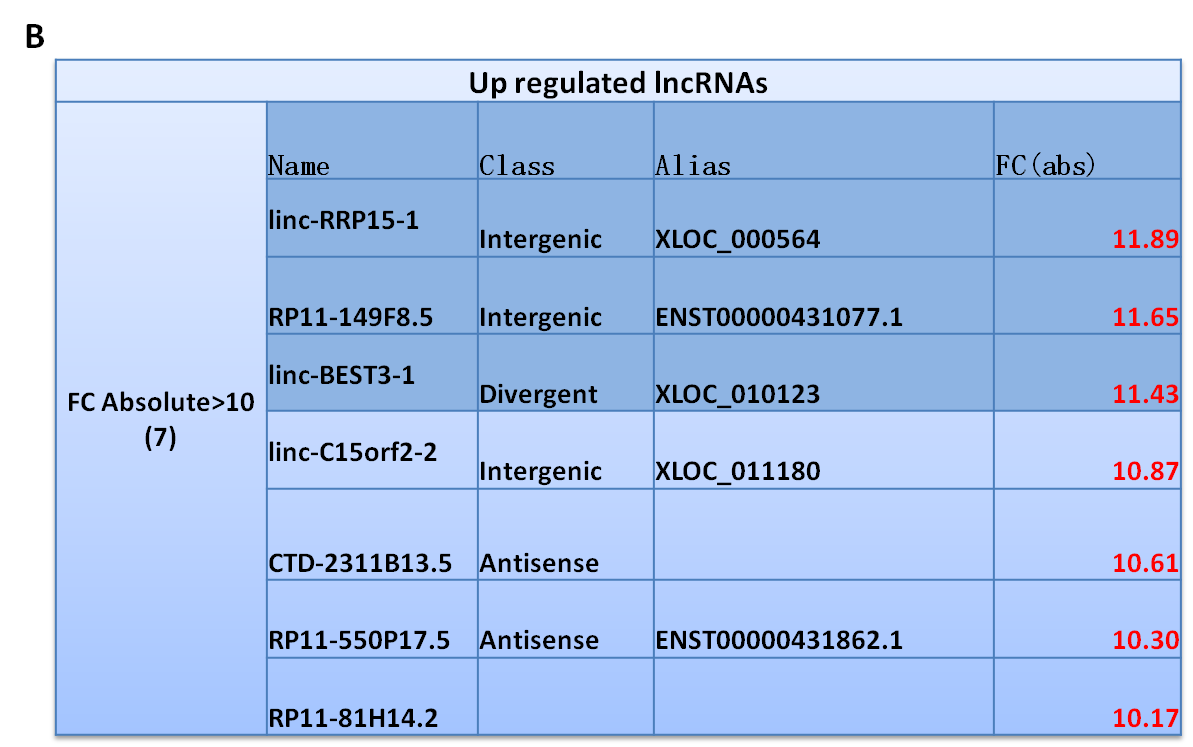


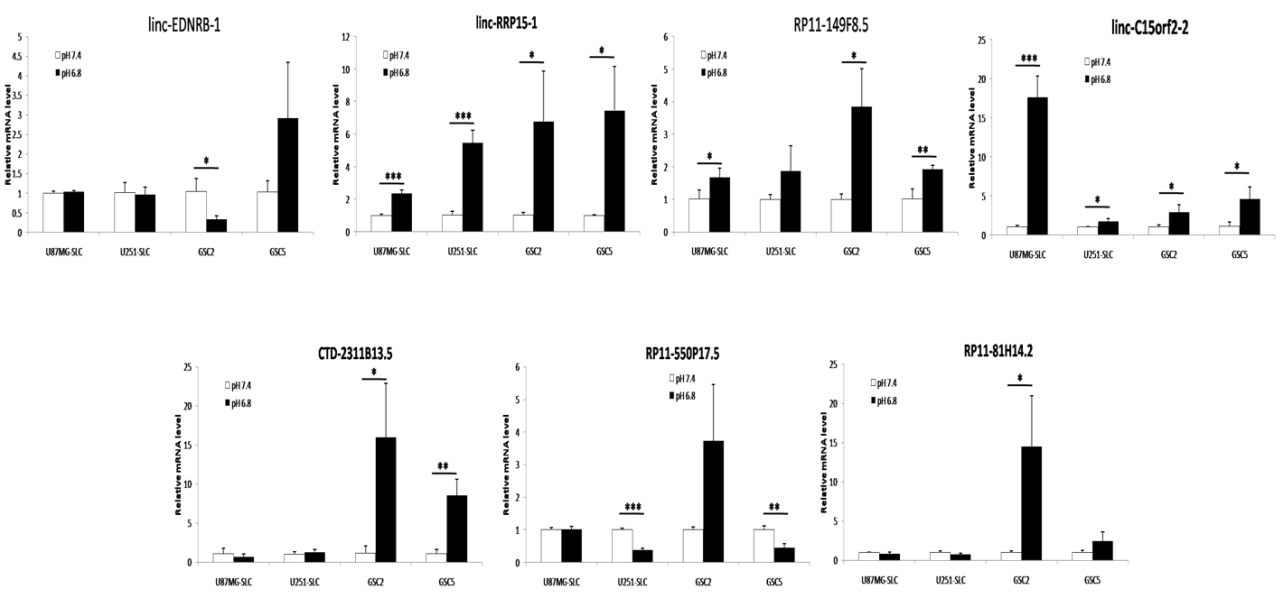


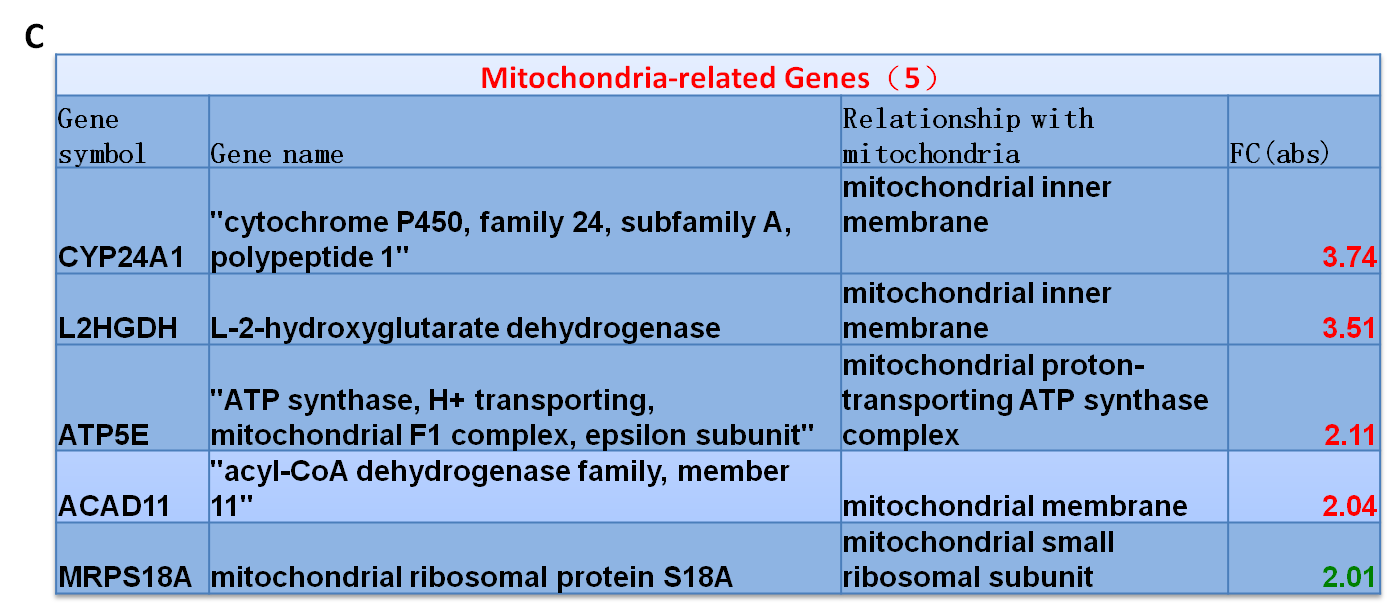


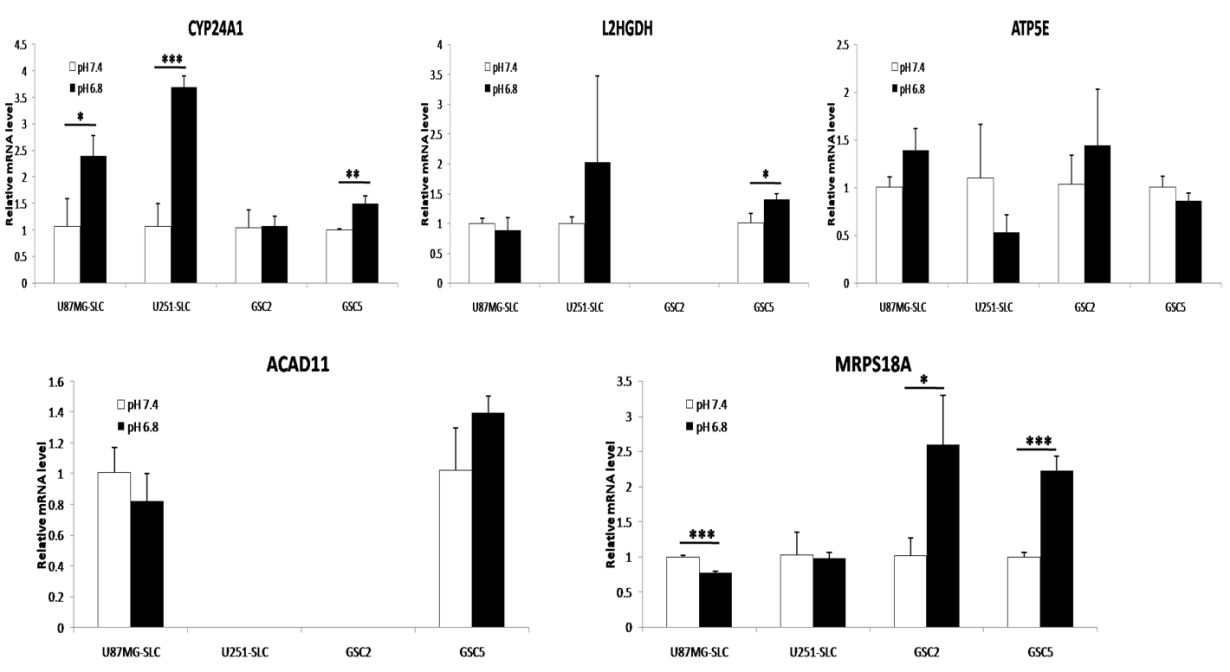


For A-C, upper pannel: introduction of the selected 7 genes (A), 7 lncRNAs (B), 5 mitochondrial function related genes (C). bottom pannel: the relative RNA level of the selected genes and lncRNAs as described in the above tables in U87MG-SLC, U251-SLC, GSC2 and GSC5 cells under pH7.4 or pH6.8 conditions (A, B and C). * *P* < 0.05, ** *P* < 0.01, *** *P* < 0.001, Student’s t-test.

**Fig.S3 Analysis the influence of 5 candidates on the stemness and mitochondrial respiration of SLCs.**

**
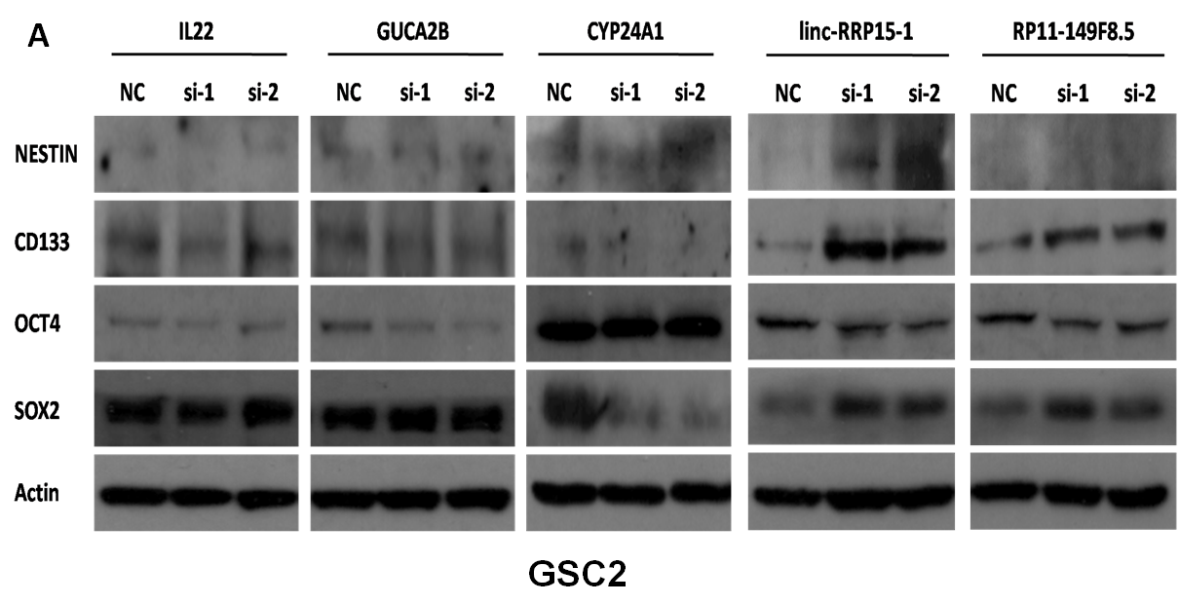
**

**
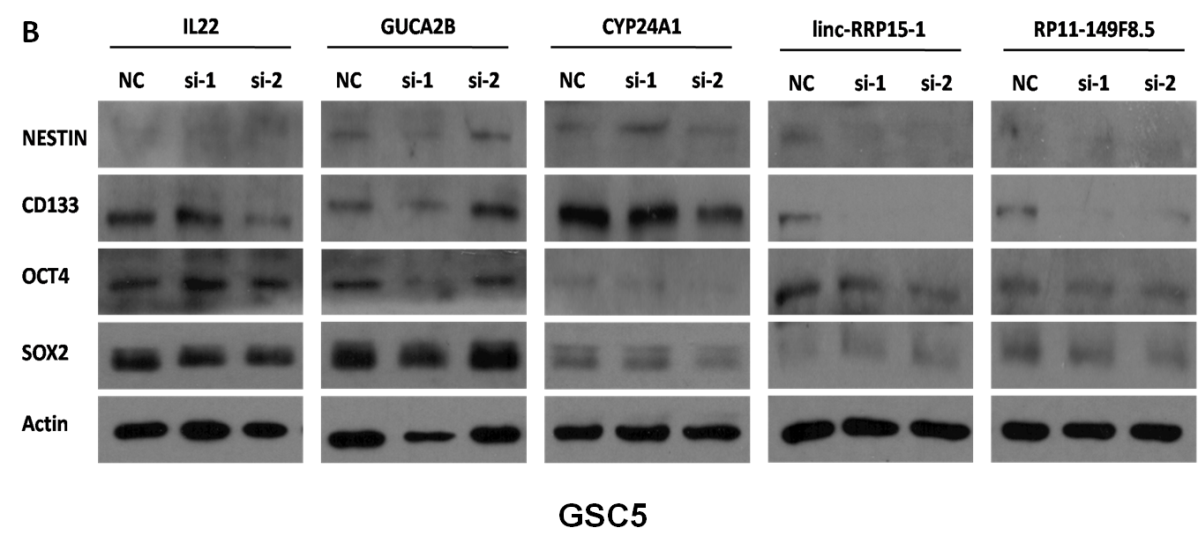
**

For A-B, Immunoblotting of the expression of stemness markers NESTIN, CD133, OCT4 and SOX2 in GSC2 and GSC5 cells that transfected with targeting siRNA of IL22, GUCA2B, CYP24A1 and lncRNA RP11-149 f8. 5, linc-RRP15-1.


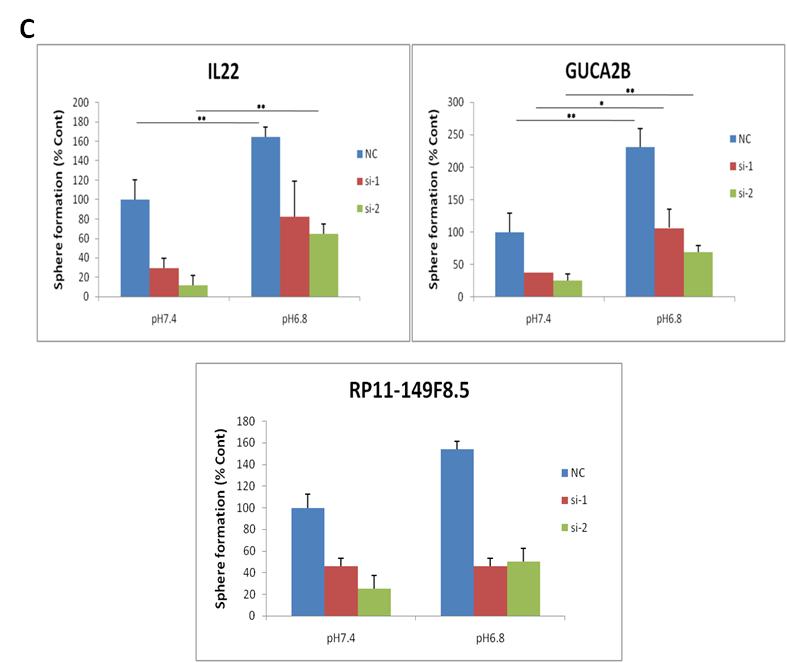


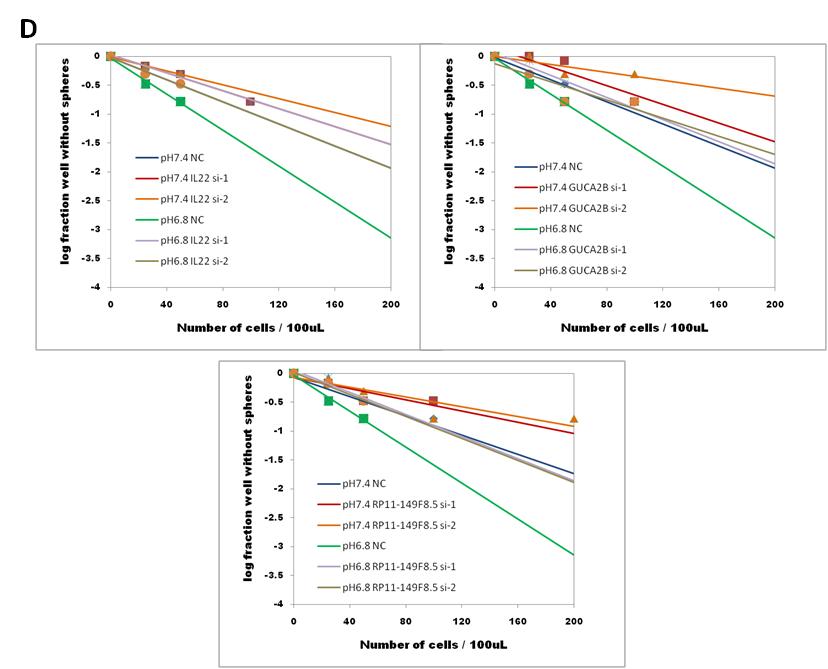


**For C-D,** self-renewal ability of SLCs while knockdown of 3 candidates under pH7.4 or pH6.8 culture conditions. Neurosphere formation assay showed the number of neurospheres (diameters larger than 50 µm) formed from U251-SLCs that transfected with targeting siRNA of IL22, GUCA2B and lncRNA RP11-149 f8. 5 (C), * *P* < 0.05, ** *P* < 0.01, *** *P* < 0.001, Student’s t-test. Limiting dilution assay of pH7.4-treated and pH6.8-treated U251-SLCs that knockdown of IL22, GUCA2B and lncRNA RP11-149 f8. 5. Cells were diluted into 200, 100, 50,25 and 0 per 100μl, wells not containing spheres (Diameter that larger than 50 μm) for each cell plating density was calculated after 2 weeks(D).


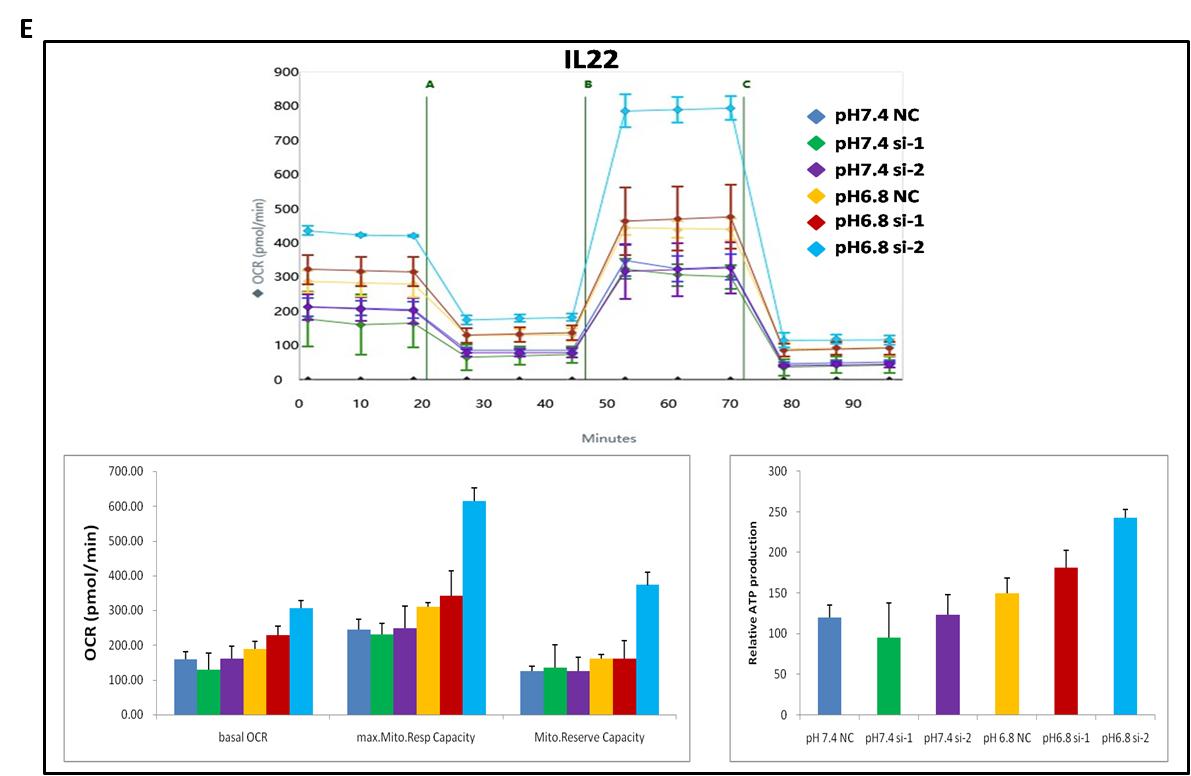


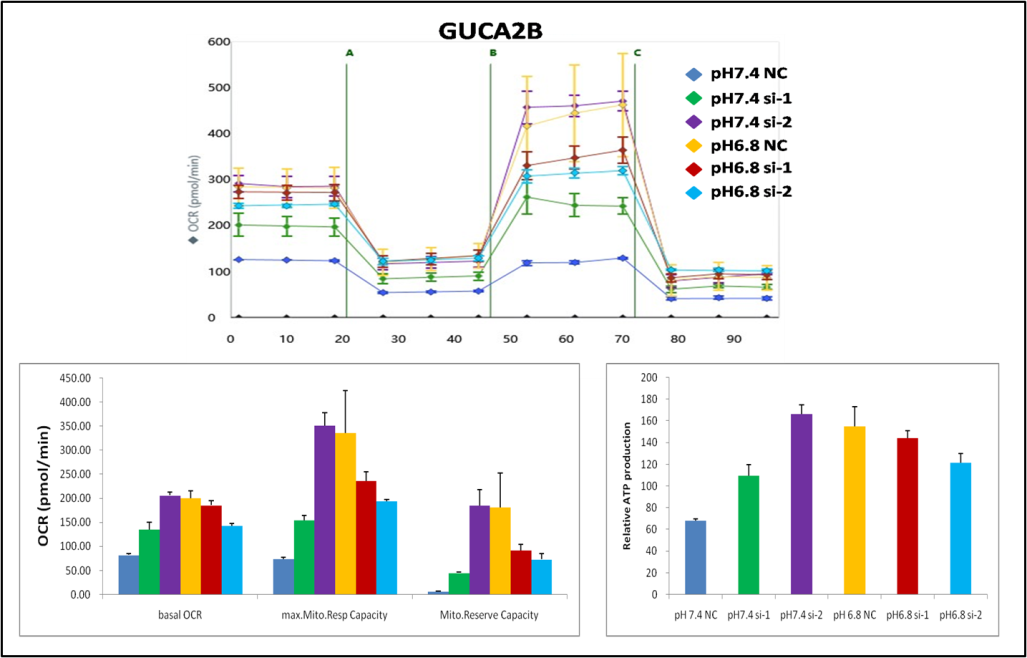


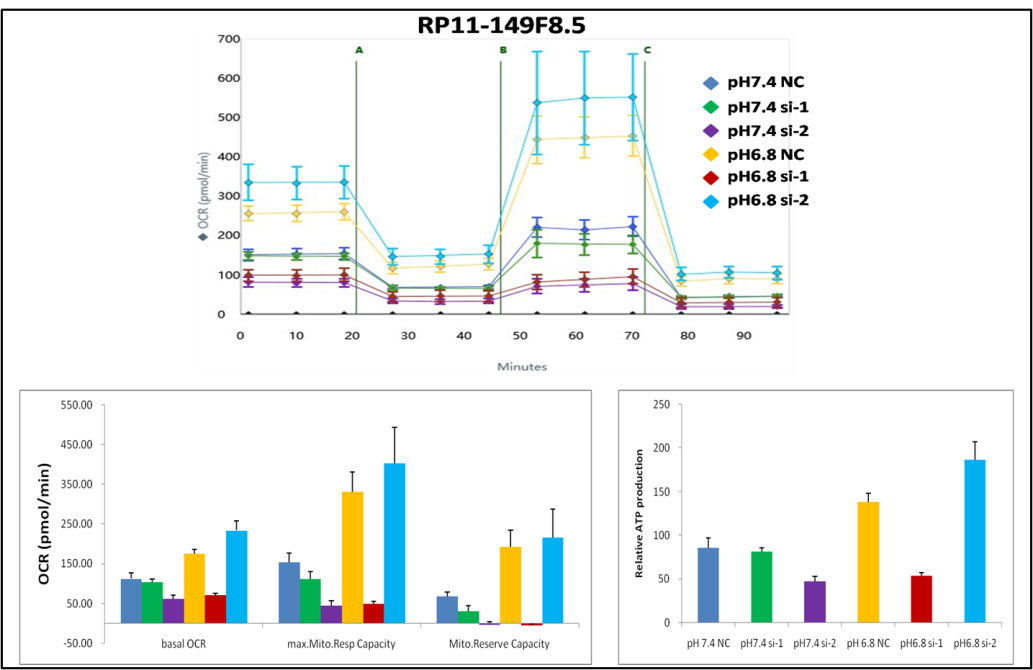


**E.** Respiration of mitochondria in U251-SLCs that knockdown of IL22, GUCA2B and lncRNA RP11-149F8. 5 under pH7.4 or pH6.8 culture conditions and treated with oligomycin (named as ‘‘A’’), FCCP (named as ‘‘B’’), Antimycin A and Rotenone (named as ‘‘C’’). Oxygen consumption rate of basal respiration (basal OCR), maximal respiration (max. Mito. Resp Capacity), spare respiratory capacity (Mito.Reserve Capacity) and ATP production were shown.

**Fig.S4 1α,25(OH)_2_D_3_ inhibited the stemness of U251-SLCs under acidosis.**


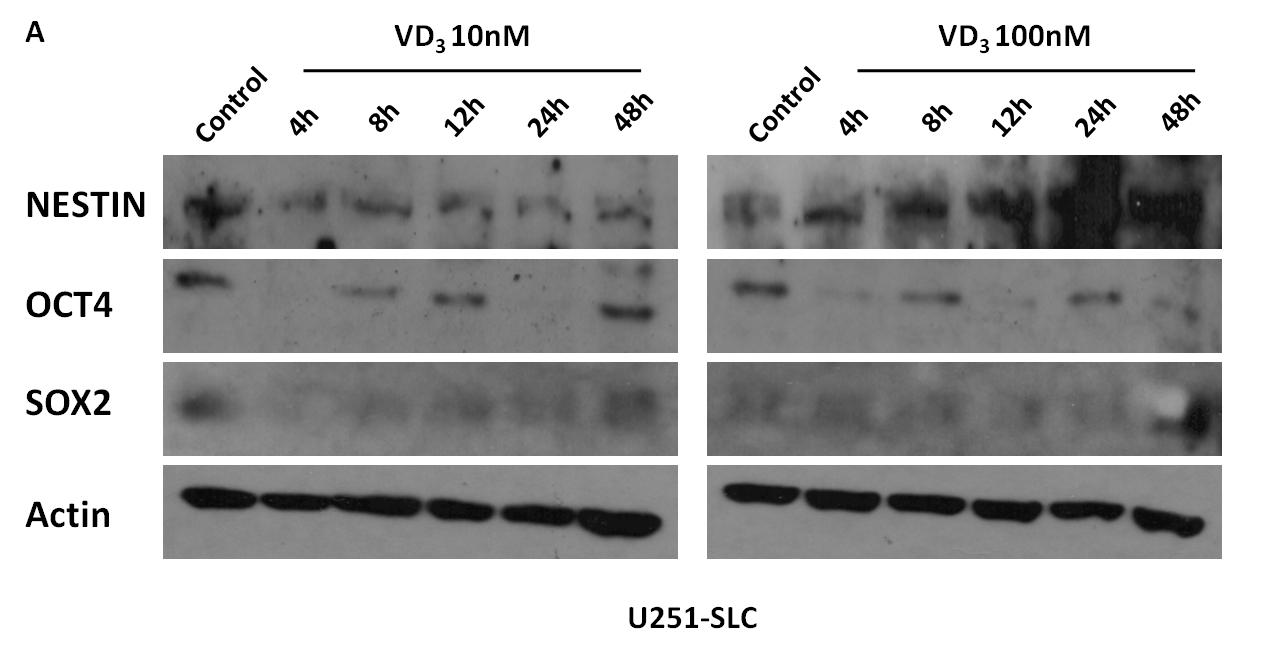


1. Immunoblotting of the expression of stemness markers NESTIN, OCT4 and SOX2 in U251-SLCs that treated with 1α,25(OH)_2_D_3_ 10nM or 100nM for 4h, 8h, 12h, 24h and 48h.

**
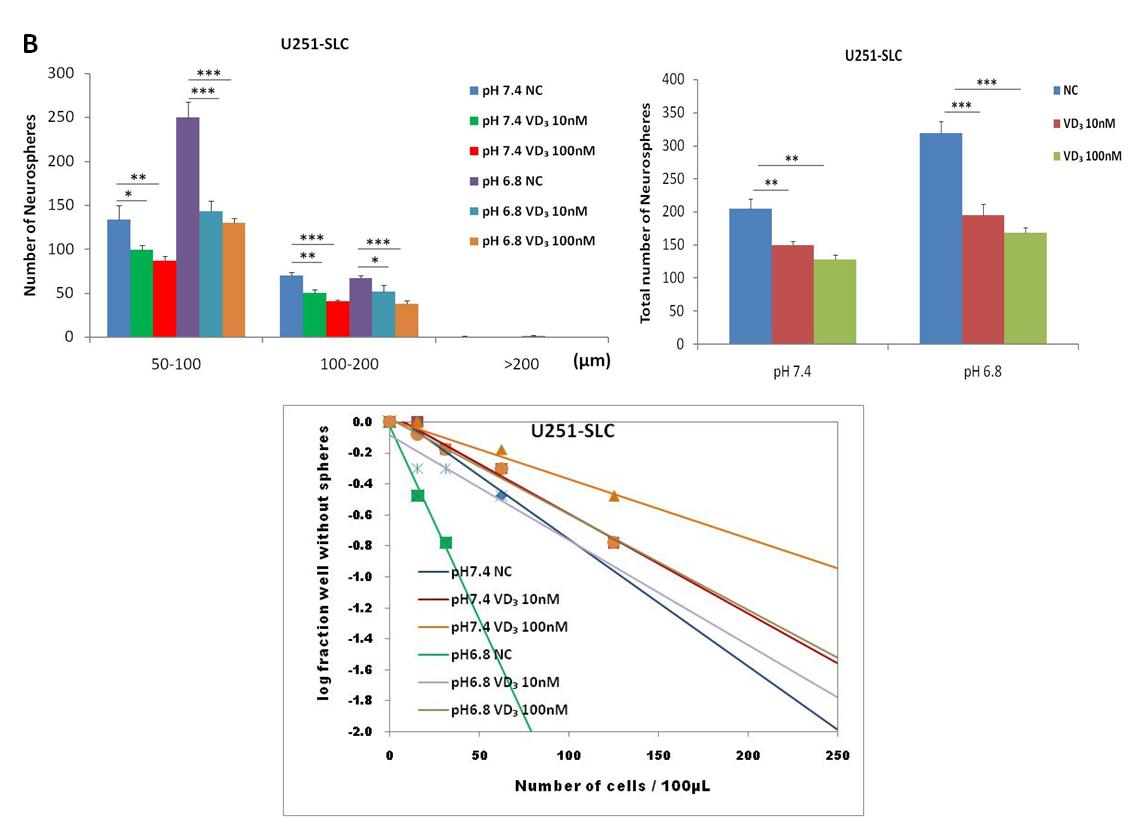
**

1. Neurosphere formation assay showed the number of neurospheres (diameters larger than 50 µm) formed from U251-SLCs that treated with 1α,25(OH)_2_D_3_ 10nM or 100nM under pH7.4 or pH6.8 culture conditions (upper panel), * *P* < 0.05, ** *P* < 0.01, *** *P* < 0.001, Student’s t-test. Limiting dilution assay of pH7.4-treated and pH6.8-treated GSC2 and U251-SLCs were diluted into 250, 125, 62.5, 31.25, 15.625 and 0 per 100μl that treated with 1α,25(OH)_2_D_3_ 10nM or 100nM. Wells not containing spheres (Diameter that larger than 50 μm) for each cell plating density was calculated after 2 weeks (bottom panel).

**Fig.S5 1α,25(OH)_2_D_3_ impaired the mitochondrial respiration of SLCs in acidic condition.**

**
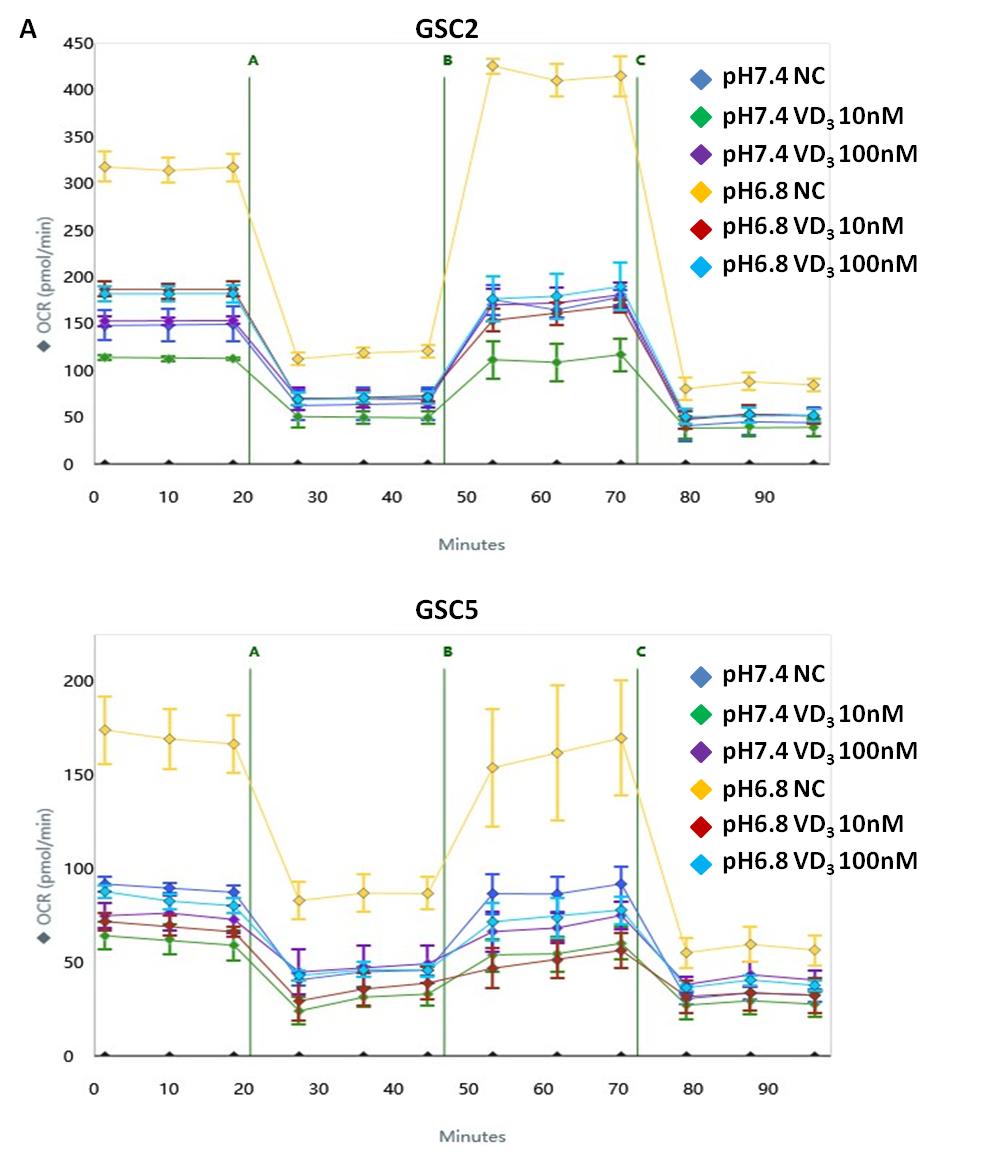
**

A. Sequential compound injections measure the oxygen consumption rate (OCR) of GSC2 and GSC5 cells that treated with 1α,25(OH)_2_D_3_ 10nM or 100nM for 4h under pH7.4 or pH6.8 culture conditions. ‘‘A’’ presents the injection of oligomycin, ‘‘B’’ presents the injection of FCCP, ‘‘C’’ presents the injection of antimycin A and rotenone.

**Fig.S6 CYP24A1 was highly expressed in high grade glioma tissues.**


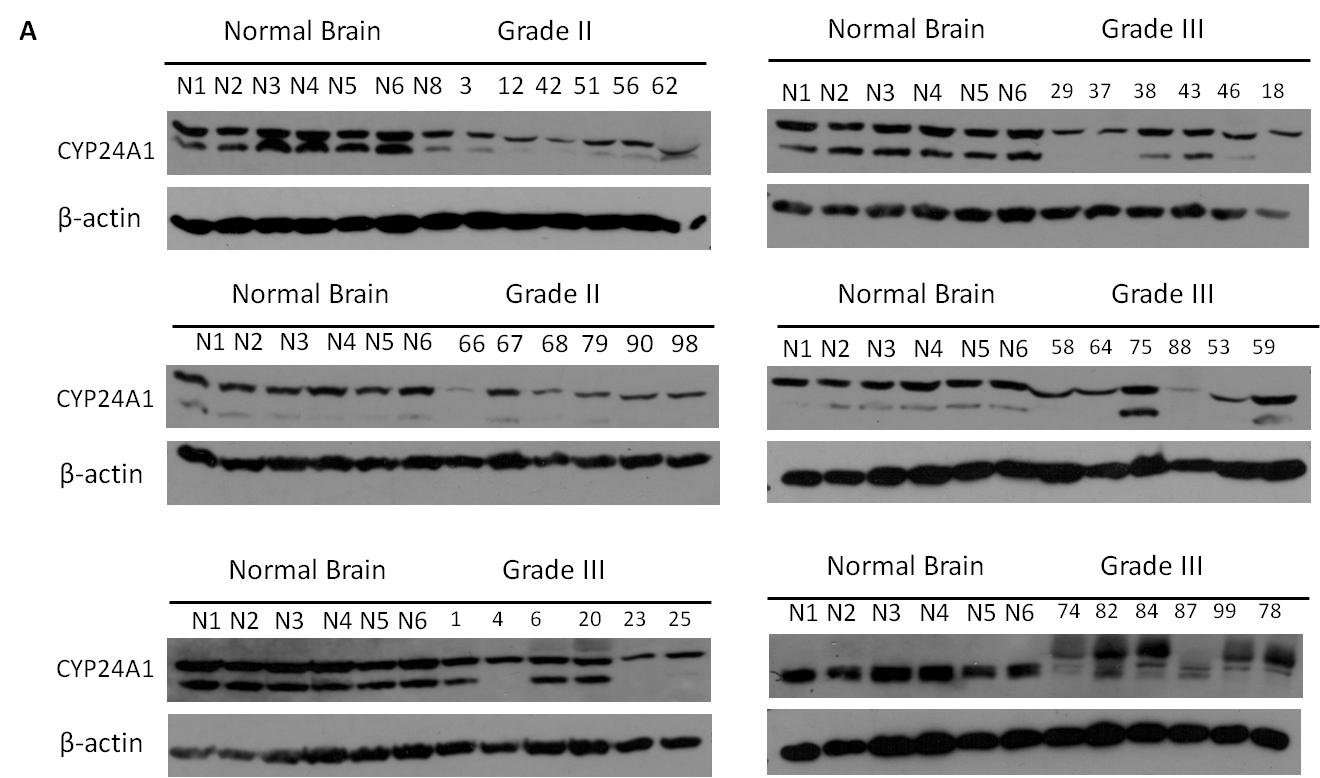


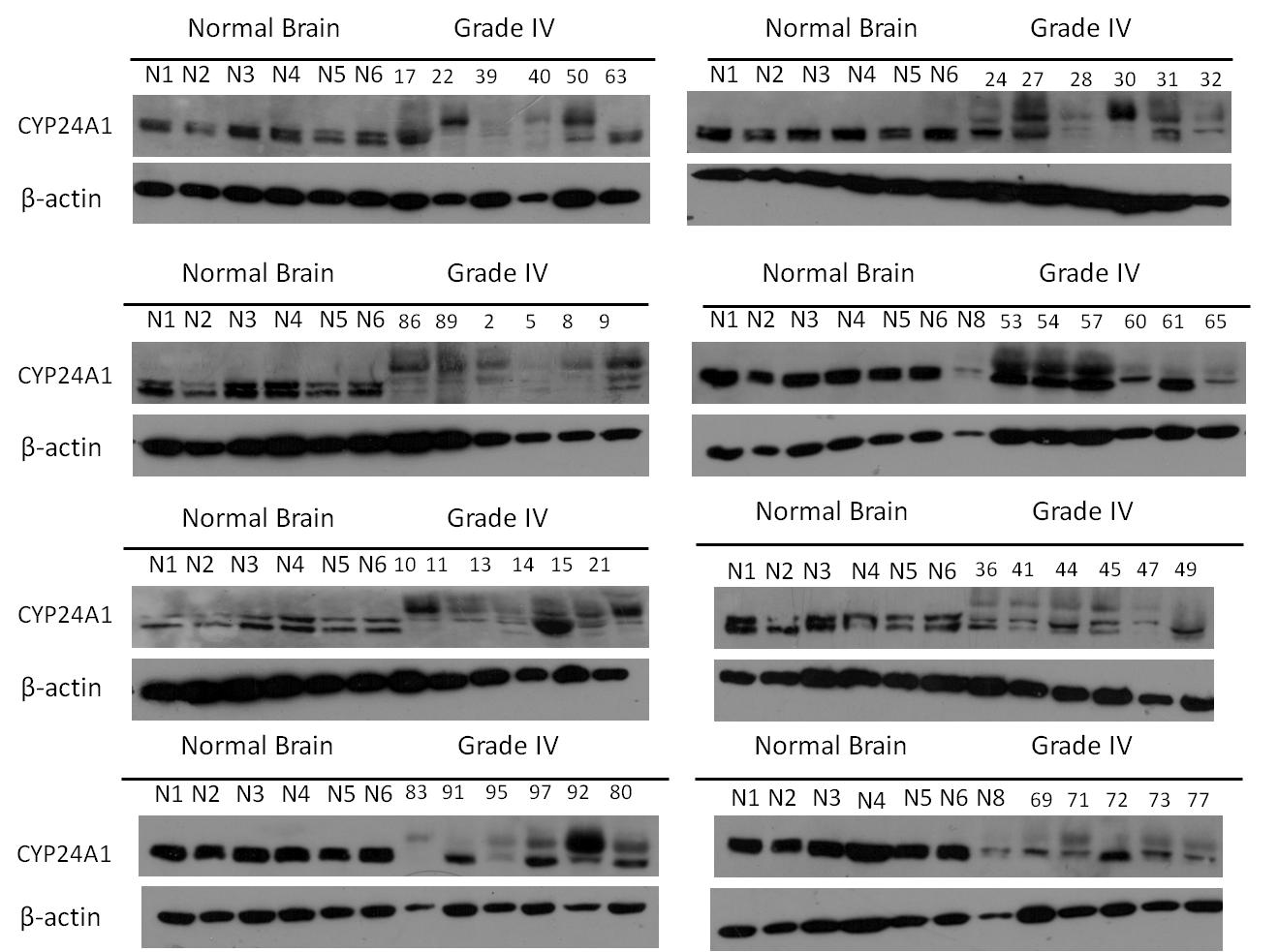


1. Images of CYP24A1 expression in 7 normal brain tissues and in 83 glioma tissues (12 grade II, 24 grade III, and 47 grade IV glioma tissues) as described in Figure 1B.

**B**

**
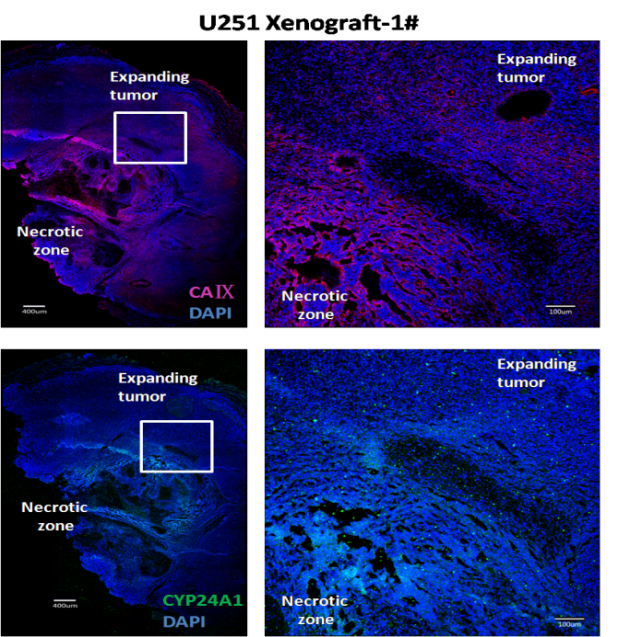

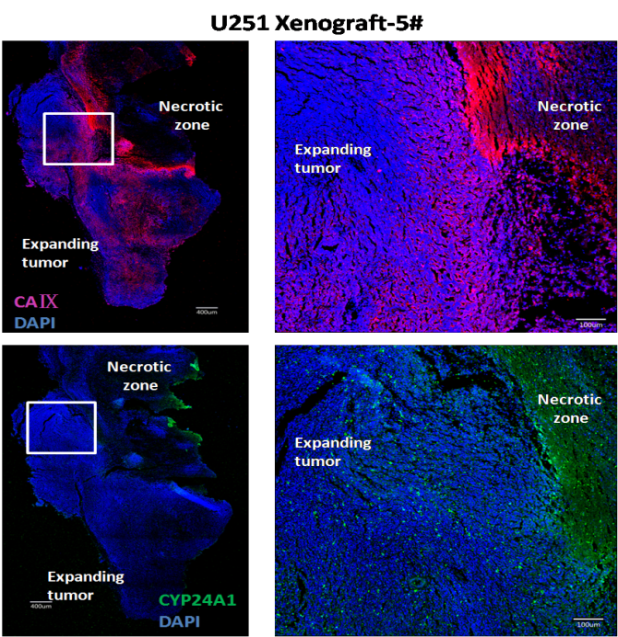
**

**
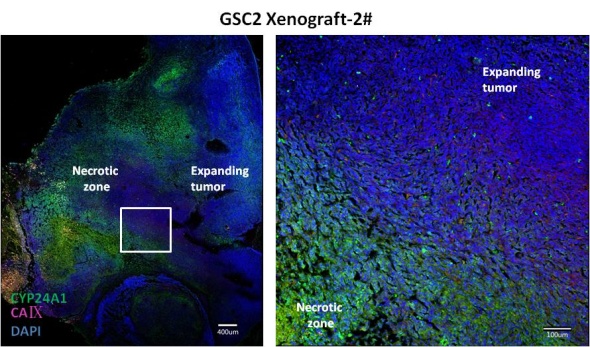

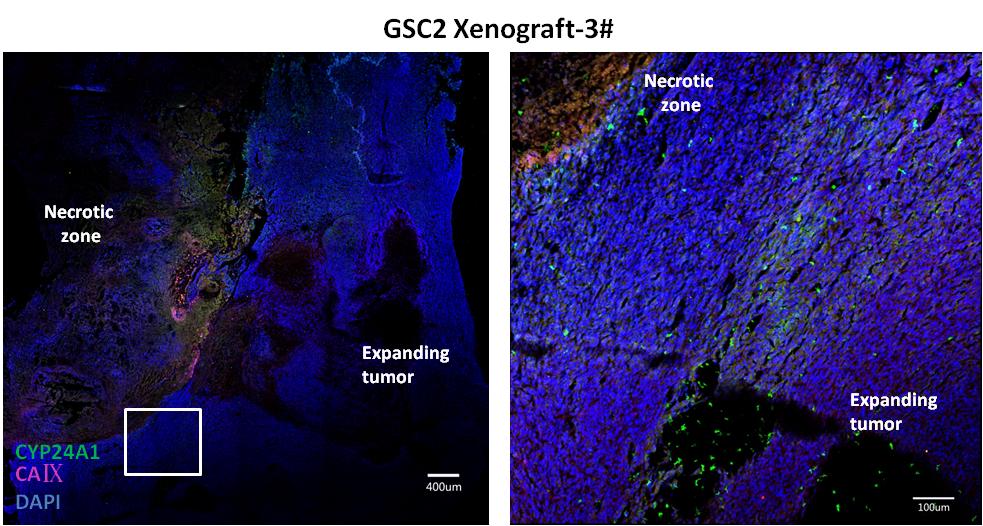

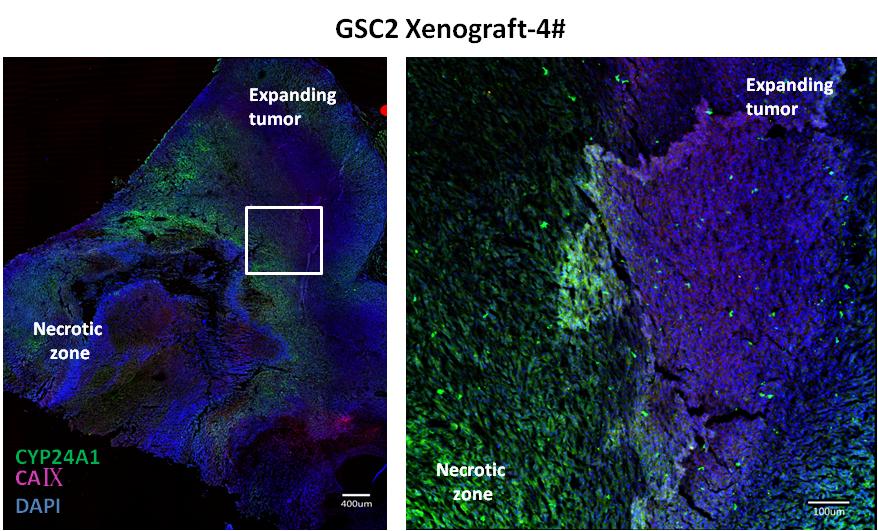
**

**
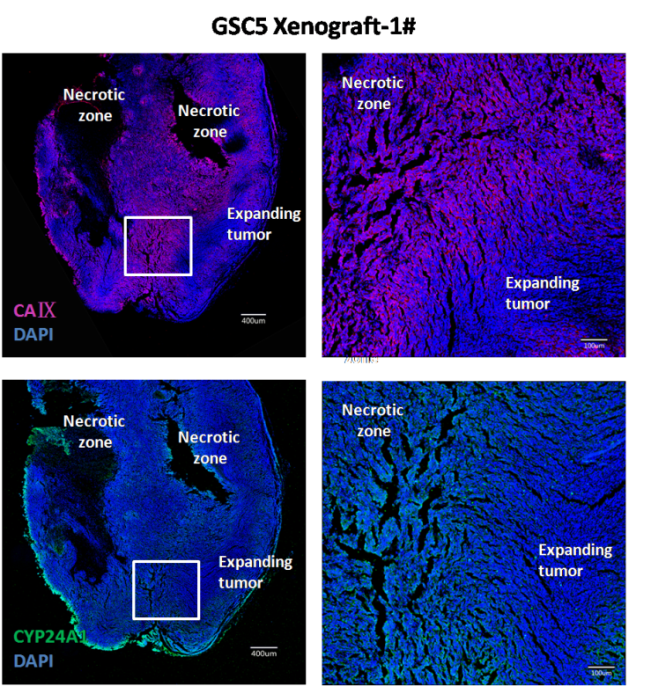

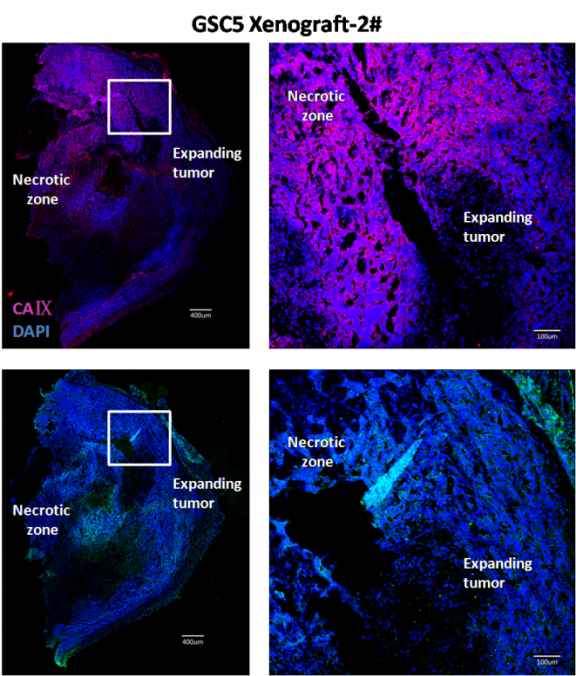
**

1. Immunofluorescence analysis of CYP24A1 (green) and carbonic anhydrase IX (CA IX, red) merged with nuclear DAPI staining (blue) in 2 xenografts developed from U251 cells, 3 xenografts developed from GSC2 cells and 2 xenografts developed from GSC5 cells. (Bar = 400 μm, left; Bar = 100 μm, right).

**Fig.S7 The expression of CYP27A1, CYP27B1 in high grade glioma tissues.**

**A**


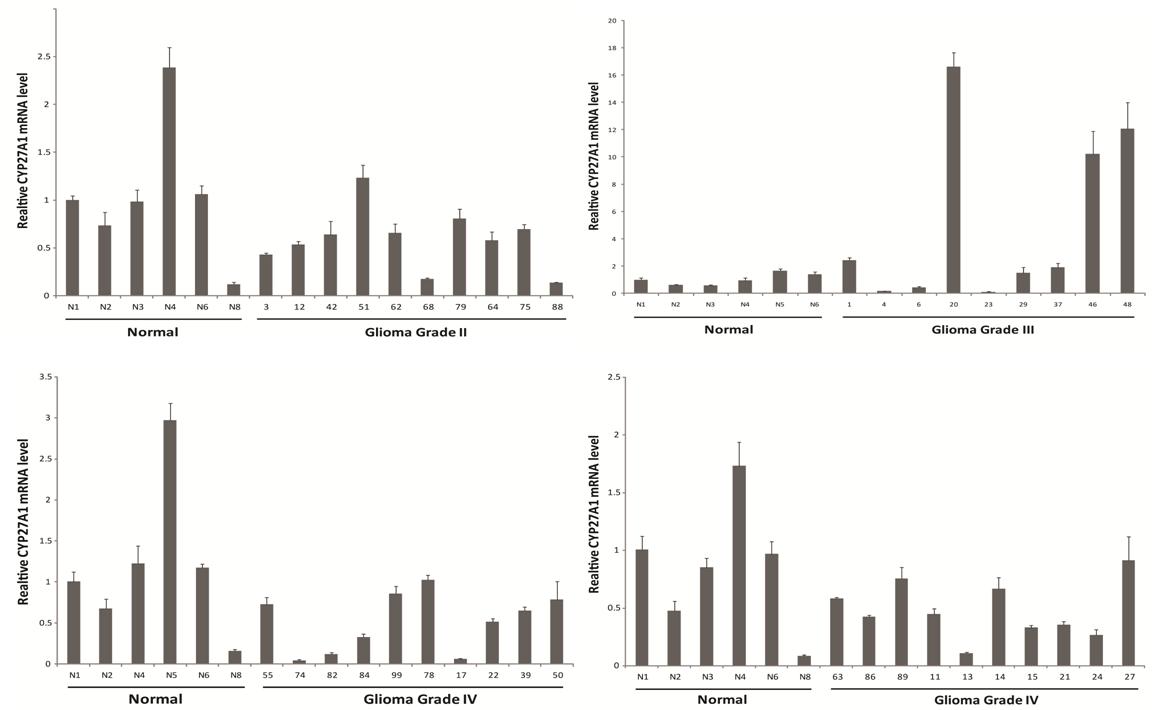


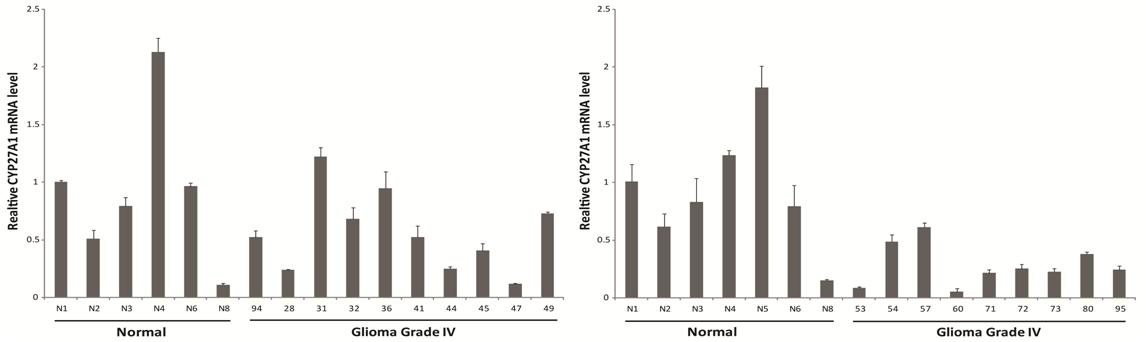


A. The relative CYP27A1 mRNA levels in 7 control brain tissues and in 10 grade II, 9 grade III, and 39 grade IV glioma tissues; actin was used as a loading control.

**B**

**
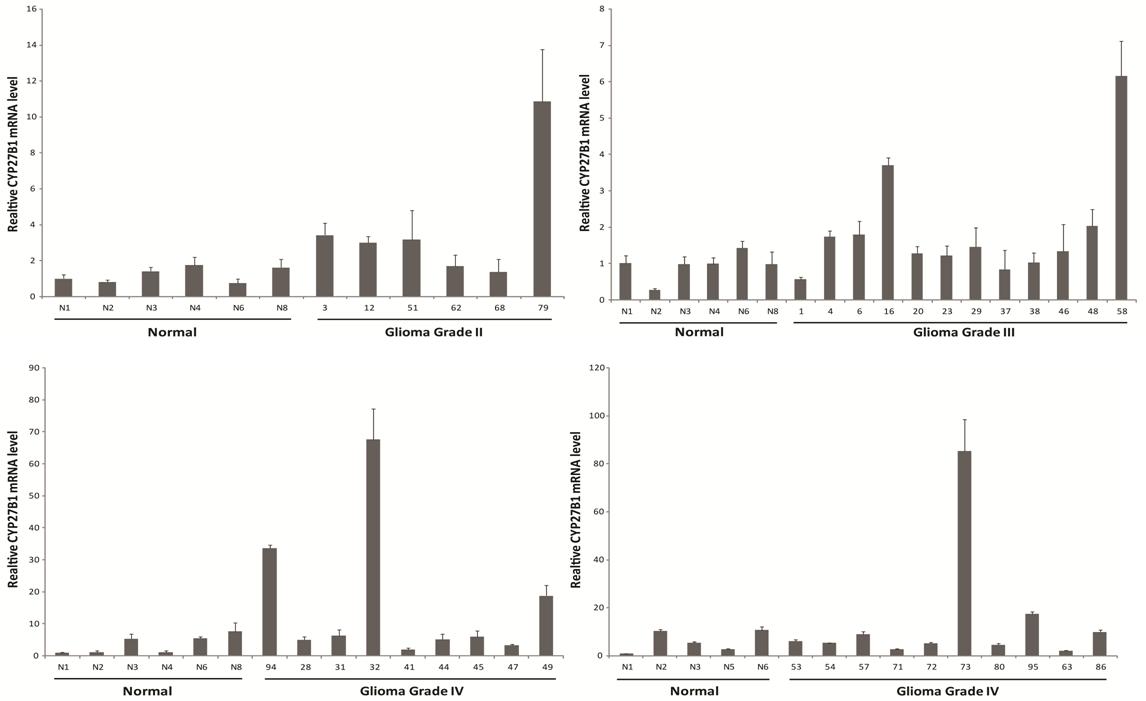

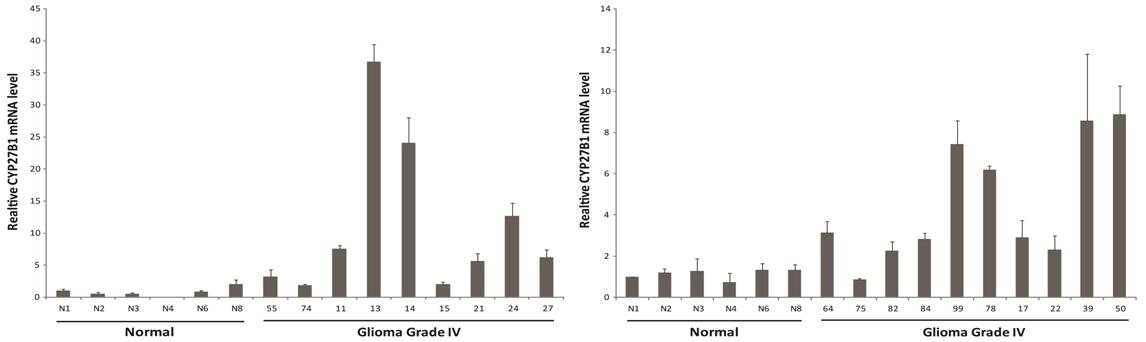
**

B. The relative CYP27B1 mRNA levels in 7 control brain tissues and in 6 grade II, 12 grade III, and 38 grade IV glioma tissues; actin was used as a loading control.
